# Steroid-Functionalized Imidazolium Salts with an Extended Spectrum of Antifungal and Antibacterial Activity

**DOI:** 10.3390/ijms222212180

**Published:** 2021-11-10

**Authors:** Marta Malinowska, Diana Sawicka, Katarzyna Niemirowicz-Laskowska, Przemysław Wielgat, Halina Car, Tomasz Hauschild, Agnieszka Hryniewicka

**Affiliations:** 1 Department of Organic Chemistry, Faculty of Chemistry, University of Bialystok, Ciołkowskiego, 1K, 15-245 Białystok, Poland; 2 Department of Experimental Pharmacology, Faculty of Health Sciences, Medical University of Bialystok, Szpitalna, 37, 15-295 Białystok, Poland; diana.sawicka@umb.edu.pl (D.S.); katarzyna.niemirowicz@umb.edu.pl (K.N.-L.); hcar@umb.edu.pl (H.C.); 3 Department of Clinical Pharmacology, Faculty of Medicine with the Division of Dentistry and Division of Medical Education in English, Medical University of Bialystok, Waszyngtona, 15A, 15-274 Białystok, Poland; przemyslaw.wielgat@umb.edu.pl; 4 Department of Microbiology and Biotechnology, Faculty of Biology, University of Bialystok, Ciołkowskiego, 1J, 15-245 Białystok, Poland; thausch@uwb.edu.pl; 5 Department of Organic Chemistry, Faculty of Pharmacy with the Division of Laboratory Medicine, Medical University of Bialystok, Mickiewicza, 2A, 15-222 Bialystok, Poland

**Keywords:** imidazolium salts, steroid, antifungal activity, antibacterial activity

## Abstract

It is established that high rates of morbidity and mortality caused by fungal infections are related to the current limited number of antifungal drugs and the toxicity of these agents. Imidazolium salts as azole derivatives can be successfully used in the treatment of fungal infections in humans. Steroid-functionalized imidazolium salts were synthesized using a new, more efficient method. As a result, 20 salts were obtained with high yields, 12 of which were synthesized and characterized for the first time. They were derivatives of lithocholic acid and 3-oxo-23,24-dinorchol-4-ene-22-al and were fully characterized by ^1^H and ^13^C nuclear magnetic resonance (NMR), infrared spectroscopy (IR), and high resolution mass spectrometry (HRMS). Due to the excellent activity against bacteria and *Candida albicans*, new research was extended to include tests on five species of pathogenic fungi and molds: *Aspergillus niger* ATCC 16888, *Aspergillus fumigatus* ATCC 204305, *Trichophyton mentagrophytes* ATCC 9533, *Cryptococcus neoformans* ATCC 14116, and *Microsporum canis* ATCC 11621. The results showed that the new salts are almost universal antifungal agents and have a broad spectrum of activity against other human pathogens. To initially assess the safety of the synthesized salts, hemocompatibility with host cells and cytotoxicity were also examined. No toxicity was observed at the concentration at which the compounds were active against pathogens.

## 1. Introduction

Recently published data has indicated that the high rates of morbidity and mortality from fungal infections are related to the current limited number of antifungal drugs and the toxicity of the agents [1]. The most important challenge is the identification of novel drug targets due to the many similarities between fungal and human cells [2]. The Food and Drug Administration (FDA) have approved four classes of antifungal agents: azoles, polyenes, echinocandins, and flucytosine. In effect, to address the aforementioned problems, researchers have attempted to find a strategy to improve treatment using various approaches. One of these is the modification of the chemical structure of traditional antifungals. To date, it has been established that the above approaches can significantly enhance their activity and improve pharmacokinetic parameters [3]. Another important aspect is the development of new formulations for antifungal agents; however, many years are required from the discovery of a new antifungal drug to clinical use [4]. As indicated by Roemer et al., even with these newest therapies, the clinical outcomes for most invasive fungal infections are far from ideal [5]. It is established that there is an increasing rate of infections caused by species of molds, or more common organisms such as *Candida* spp., for which there is no reliable medical therapy due to the acquired resistance to currently used drugs [6]. In effect, it seems that the rate of antifungal drug discovery has not kept up with the clinical needs. It should be highlighted that patients at risk of invasive fungal infections may also be at risk of developing serious bacterial infections, or vice versa [7]. It has been established that long-term antibiotic-based therapy causes uncontrolled fungal growth. It has also been suggested that fungal-bacterial interactions often occur during infections [8]. The interaction of fungi and bacteria can be realized via multiple mechanisms and cause different effects on the host. For example, the antagonistic interaction often limits microbial virulence, while synergistic relationships markedly potentiate pathogenesis [9,10]. Recent reports have shown that up to 38% of candidemia cases were mixed infections [11]. In effect, antibacterial/antifungal agents might be used for both prophylactic and therapeutic purposes in parallel or sequentially [12]. The ideal solution will be the application of a single therapeutic agent that will have both antibacterial and antifungal properties. Therefore, there is an urgent need to develop new agents that will possess a broad spectrum of action in the treatment or prevention against life-threatening infections [1].

**Figure 1:**
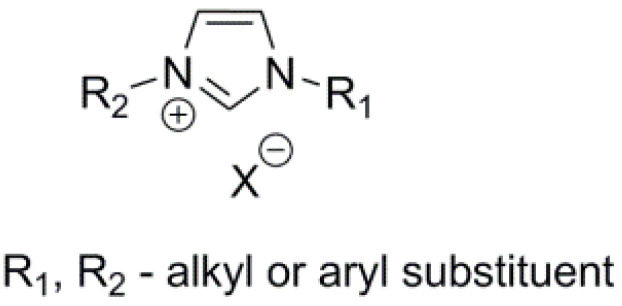
The general formula of imidazolium salts.

Azoles inhibit the activity of 14-α-sterol demethylase, limiting the biosynthesis of ergosterol [13]. Imidazolium salts (Figure 1) are azole-based compounds that can be used as antifungal agents [14,15]. They are biologically significant compounds derived from imidazole with two substituted nitrogen atoms. The imidazole ring is present in many structures of the human body that play an essential role in its functioning [16]. Imidazole can bind metals as a ligand or form a hydrogen bond with drugs and proteins [17,18]. Imidazolium salts can interact electrostatically with biological systems [19,20]. In addition, molecular dynamics simulations revealed that the proteins create more salt bridges in the presence of imidazolium salts, which turned out to be stronger than in a pure aqueous solution [21]. One of the most important features of imidazolium salts is their flexibility, allowing the structure to be properly designed to achieve the desired properties. It has been proved that an independent modification of the structure of cation or anion gives the obtained salt molecule different properties [22].

Imidazolium salts are not only important for their biological activity; they have also found application in organic synthesis as ionic liquids that can be used as electrolytes or solvents in green chemistry [23,24]. Moreover, these compounds are precursors of *N*-heterocyclic carbenes (NHCs), which can be obtained after deprotonation of the salts at the C2 position [25,26]. NHCs are a very useful tool in modern organic synthesis, mainly due to their ability to bind most transition metals. Metal complexes with NHCs are homogeneous catalysts for many reactions (mainly as ruthenium and palladium complexes) [27,28]; additionally, NHCs themselves can be used as organocatalysts [29,30,31]. Moreover, it has been shown that NHC-metal complexes exhibit antibacterial and antitumor activity, especially when the NHCs are silver- or gold-bound ligands [32,33].

**Figure 2:**
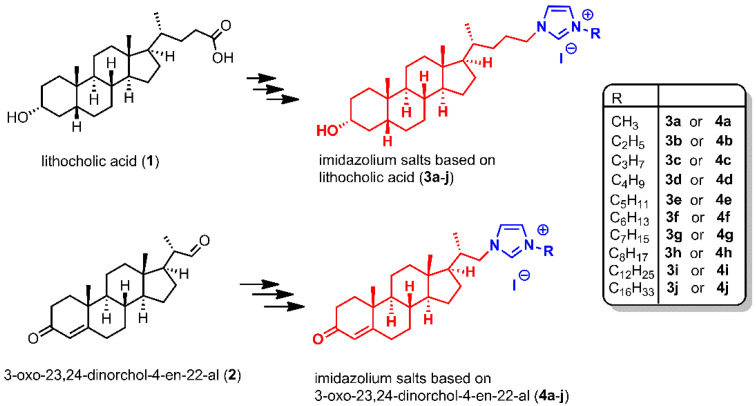
Two series of imidazolium salts tested in this study.

We recently described the synthesis and antimicrobial activity of two series of steroid-based imidazolium salts [34], which were derived from lithocholic acid and one of lithocholic acid metabolites [35] 3-oxo-23,24-dinorchol-4-en-22-al (**1** and **2**, Figure 2). The new salts had a steroid substituent on one nitrogen atom and an alkyl group on the other. As a result, eight asymmetric steroid-based salts with different substituents: methyl, ethyl, pentyl, and hexyl (**3a**, **3b**, **3e**, **3f** and **4a**, **4b**, **4e**, **4f**, Figure 2) were obtained. Due to the biological activity of the steroid in combination with its large enantiomerically pure backbone, the properties of these salts proved to be of great interest. Additionally, in preliminary studies, the activity of *N*-steroid-substituted imidazoles (**5** and **6**) and their protonated form were determined. These tests have shown that both quaternization and the presence of an alkyl substituent on the nitrogen atom are essential to obtain the desired activity. The type of counter-ion also plays a role in the antifungal activity, and the iodide anion has proved to be the best choice. Therefore, our further work has focused on steroid imidazolium iodides. Initial screening of the above-mentioned salts showed that they have promising activity against *C. albicans*, exceeding the antifungal activity of the commonly used antifungal agents [34].

This article is a continuation of this interesting research, extending it to other steroid-based salts. Therefore, twelve new imidazolium salts were obtained with different side-chain lengths (with 3, 4, 7, 8, 12, and 16 carbon atoms). Biological evaluation of the new salts was performed. Moreover, special emphasis in our research was placed on determining the antifungal activity against the various pathogenic fungi of all steroid-based imidazolium salts (a total of 20 compounds—both previously described and new ones). Moreover, the in vitro hemolytic activity on human red blood cells was assessed and the fibroblast cytotoxicity was determined.

## 2. Results and Discussion

### 2.1. Synthesis of Imidazolium Salts

**Scheme 1:**
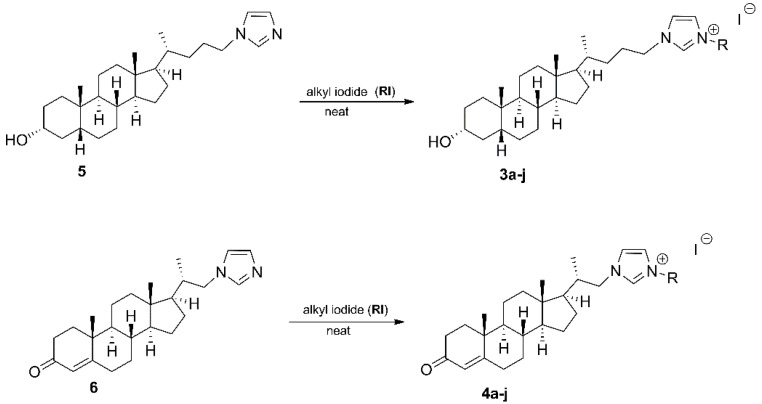
Synthesis of imidazolium salts **3a**–**j** and **4a**–**j**.

The steroid-based salts with the shortest chain lengths (**3a**,**b** and **4a**,**b**, Figure 2) were prepared from *N*-steroid-substituted imidazole (**5** and **6**, respectively) according to the previously reported methodology [34]. The procedure was modified for the synthesis of salts with longer side chains (Scheme 1). By using high-boiling alkyl iodides (propyl, butyl, pentyl, hexyl, heptyl, octyl, dodecyl, and hexadecyl iodides), the reaction could be carried out at an elevated temperature (80 °C or 100 °C). This allows the reaction time to be shortened (e.g., from 24 to 2 h or even 15 min). Moreover, the yield of these reactions exceeded 70%, with only a few exceptions (**3b**, **3i**,**j**, and **4j**). The new method made it possible to obtain four known salts (**3e**,**f** and **4e**,**f**) with a twice higher yield than before. In the case of the **4f**, this modification allows quantitative conversion, as well as reduction of the reaction time. As a result, 20 salts of different chain lengths, depending on the alkyl iodide used, were obtained. It should be emphasized that 12 salts (**3c**,**d**, **3g**–**j**, **4c**,**d**, and **4g**–**j**, Table 1) are new compounds; their synthesis is described here for the first time.

### 2.2. Antimicrobial Studies

In the previously reported study, antimicrobial activity against *S. aureus*, *B. cereus, E. coli,* and *C. albicans* was presented for only a few steroid-based imidazolium salts [34]. Encouraged by these results, we decided to supply them with those collected for new salts (two series, total of 20 compounds). The results of minimum inhibitory concentration (MIC) experiments against bacteria are summarized in Table 2. According to the obtained results, almost all new salts have sufficient activity against tested bacterial species, especially those with heptyl and octyl substituents (**3g** and **3h**), which achieved the lowest values of MIC. It should be highlighted that **3g** and **3h** with MIC values less than or equal to 0.06 µg/mL were found to be 4-fold more effective against *S. aureus* than commercial Ampicillin. All tested lithocholic acid-based salts showed better activity against *S. aureus* than those based on 3-oxo-23,24-dinorchol-4-en-22-al. On the other hand, the salts of the second series showed better activity against *E. coli* than those of the first series. The obtained MIC values for salts **4c**–**h** are comparable to those of Ampicillin. All tested salts were inactive against *P. aeruginosa* with MIC values equal to or higher than 512 µg/mL (the results are not shown in Table 2).

The preliminary studies [34] showed the most promising effect of the salts against *C. albicans*-fungi most often isolated from immunocompromised patients. Therefore, we decided to investigate other salts against *C. albicans* (Table 3). The salts with a propyl (**4c**), butyl (**4d**), heptyl (**4g**), octyl (**4h**), and dodecyl (**4i**) substituent exhibited excellent activity that was better or equal to commercial Fluconazole or Amphotericin B. It should be stressed that salts **4c** and **4d** were found to be 8-fold more effective than those drugs.

Additionally, all compounds were tested on other fungal species—*Aspergillus niger*, *Aspergillus fumigatus*, *Trichophyton mentagrophytes*, *Cryptococcus neoformans*, and *Microsporum canis*. The MIC volumes are summarized in Table 3 and compared with commercially available antifungal drugs: Fluconazole, Amphotericin B, and Voriconazole.

**Figure 3:**
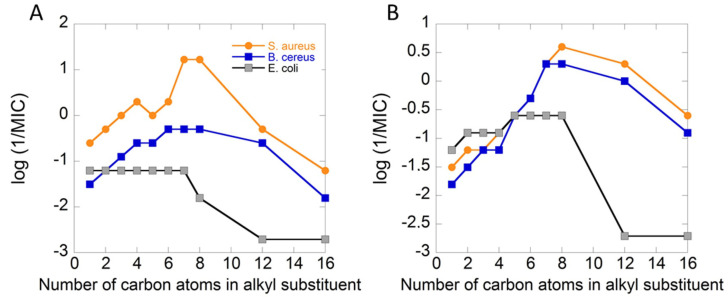
The relationship between the activity (in the log (1/MIC)) of the imidazolium salts against bacterial species and the number of carbon atoms in the alkyl chain ((**A**): the salts **3a-j**, (**B**): the salts **4a-j**).

**Figure 4:**
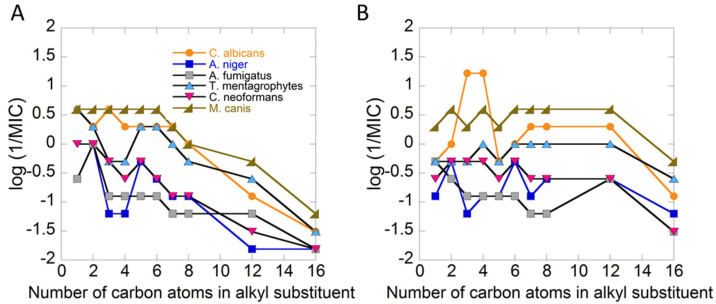
The relationship between the activity (in the log (1/MIC) of the salts against fungal species and the number of carbon atoms in the alkyl chain ((**A**): the salts **3a-j**, (**B**): the salts **4a-j**).

To better understand the relationship between the activity (in the log (1/MIC)) of the steroidal salts against microbial species and the number of carbon atoms in the alkyl chain of the salts, the results are plotted in Figure 3 and Figure 4. In general, the antibacterial activity of all salts increased in the range of carbon atoms in the alkyl chain from one to seven (except **4e** and **4f**). From eight carbon atoms in the alkyl chain, the effectiveness against human pathogens decreases in all tested compounds. This trend is the most visible for *E. coli*, where the salts with 12 and 16 carbon atoms (**3i**–**j**, and **4i**–**j**) were inactive.

In our previous study on dehydroepiandrosterone-derived imidazolium salts, we suggested that the ‘side chain effect’ may be crucial in the observed antimicrobial activity of synthesized hybrids [36]. A strong antimicrobial efficacy was observed when compounds have alkyl chain lengths greater than four and lower than twelve carbon atoms. In the present research, the highest antifungal activity is shown by lithocholic-based salts with 1–6 carbon atoms in the alkyl substituent (**3a**–**f**, except **3c** and **3d**, against *A. niger* and *T. mentagrophytes*). The highest activity is revealed by salt **3b**, which is the best candidate for antifungal therapy. In the case of the **2** derivative salts, their antifungal activity is less dependent on the length of the alkyl substituent. The most active salts are **4c** and **4d** against *C. albicans*.

### 2.3. Effect of ClogP Values on Antimicrobial Activity

Various biological processes of bioactive molecules including transportation, distribution, or metabolism depend on their lipophilic properties [37]. The bioactivity of target molecules can be predicted using the calculated lipid/water partition coefficients (ClogP) [38]. To further study the physical and chemical properties of new salts, the values of ClogP for all imidazolium cations were calculated using ChemBioOffice 2012, and the obtained results are shown in Table 4. In general, these compounds are all lipophilic (ClogP > 0). ClogP values of all compounds are raised with the increasing length of the alkyl chain. Values of ClogP ranged from 3.467 to 11.402 for lithocholic acid derivatives and from 1.863 to 9.798 for salts derived from steroid **2** (Table 4).

Based on the collected data, it can be seen that there is a certain relationship between the lipophilicity and the antifungal activity of the tested compounds. In general, the salts with lower ClogP values (<6) displayed better antimicrobial activities than compounds with high ClogP values (>8). Therefore, salts with higher lipophilicity showed poor inhibitory activity (high MIC values against bacterial or fungal species). An explanation for this may be that the higher lipophilicity of the compounds is detrimental to delivery to the binding sites in the organism. Suitable lipophilicity plays an important role in drug design.

Due to the promising antimicrobial effect of the new salts, their toxicity had to be determined to consider them as antifungal agents. For this purpose, their compatibility with the representative of host cells and fibroblast cytotoxicity have been determined.

### 2.4. Evaluation of the Hemolytic Activity of the Tested Compounds

**Figure 5:**
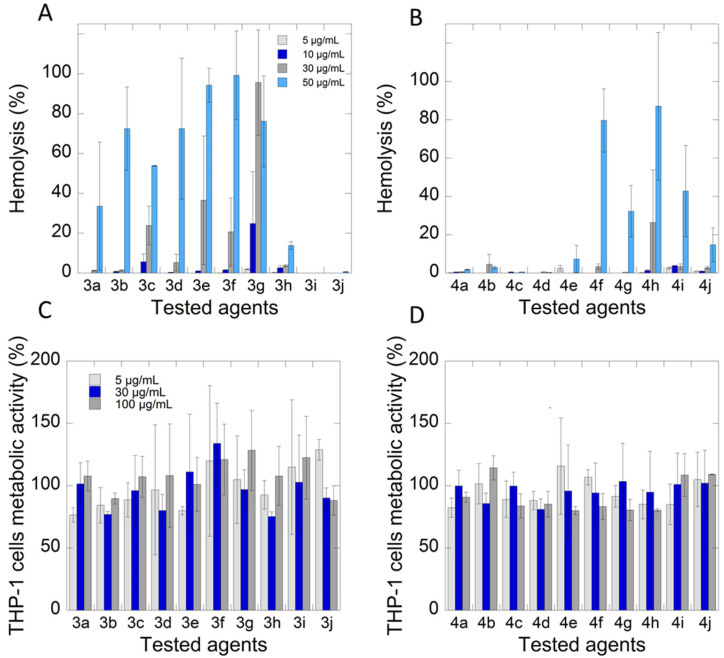
Hemocompatibility of synthesized salts. Hemolytic activity of synthesized salts **3a**–**j** (**A**) and **4a**–**j** (**B**). Metabolic activity of THP-1 cells after treatment by synthesized salts **3a**–**j** (**C**) and **4a**–**j** (**D**). The results are presented as the mean values ± SD.

One of the significant criteria for the successful in vivo use of biologically active agents is their hemocompatibility. For this purpose, the compatibility of synthesized salts was evaluated by interaction with human red blood cells for the analysis hemolysis, as well as with representatives of immune cells to determine their metabolic activity. The results showed that blood compatibility depends on the concentration and the number of carbon atoms in the alkyl substituents. To better indicate the hemolytic activity of the tested salts, a hemolytic concentration of 50 (HC50) was calculated. This is one of the most commonly used indicators of toxicity and is defined as the concentration of antimicrobial agent that caused lysis of 50% red blood cells (RBCs) [39]. As shown in Table 5, in most cases the concentration affecting 50% human RBC lysis (HC50) is greater than 50 μg/mL, which is 100–1000 fold of the MIC value. These results suggest that the proposed salts exert high antimicrobial efficacy with low hemolytic activity. The ability to induce hemolysis were indicated during antibiotic therapy, including β-lactams and polypeptides used in the treatment of infections caused by *S. aureus*, as well as in polyenes, which are used in the case of fungal infection caused by *Candida* spp. and molds. Our results indicated high antimicrobial efficacy of synthesized salts against the above-mentioned microorganism, with low hemolytic activity at a concentration that restricts the growth of the microorganisms and allows achieving a therapeutic efficacy corresponding to the MIC value and 10× the MIC value (Figure 5A,B).

In the next step of the study, we investigated the impact of salts on the metabolic activities of representatives of immune cells. The main function of monocyte/macrophage cells is to effectively protect against infection through the phagocytosis process of invading pathogens. For this purpose, our systems were evaluated in terms of cytotoxicity against monocytic THP-1 cells, which is frequently used in numerous in vitro studies, in particular, for the analyses of new therapeutic agents [40]. The results showed no apparent toxic effect after treating THP-1 cells by all tested salts used in the concentration range 5–100 µg/mL (Figure 5C,D). In effect, this may suggest that if synthesized salts are used in vivo, they will be able to maintain therapeutic efficacy without side effects.

### 2.5. Cytotoxicity Studies

**Figure 6:**
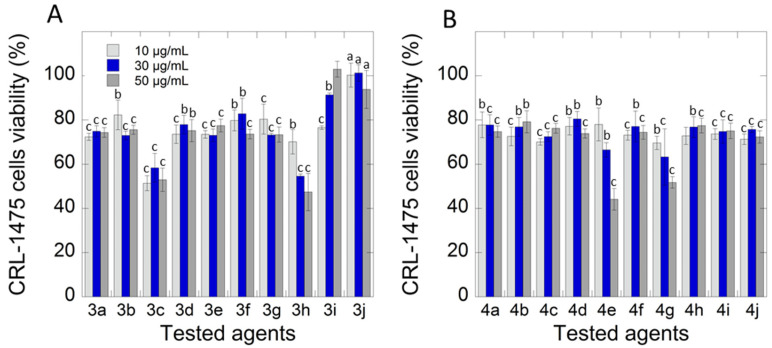
Viability of fibroblast CRL-1475 cells treated by synthesized salts **3a**–**j** (**A**) and **4a**–**j** (**B**) at concentration 10–50 µg/mL. The results are presented as the mean values + SD. a *p* < 0.05, b *p* < 0.01, c *p* < 0.001 compared to the control.

To investigate the cytotoxic effect of the salts as a drug, fibroblasts CRL-1475 cells were treated with different doses of the studied compounds. Cytotoxicity tests are considered as screening assays, used to evaluate the general toxicity of medical substances and devices. The measure of cytotoxic activity is cell viability in the presence of the test substances. For this purpose, fibroblasts are the most often used, and their main advantage is their stability and homogeneity [41]. The cytotoxicity assay indicated that the tested compounds were able to slightly inhibit the growth of fibroblast cells. There are differences in the cytotoxic effect and the structure of tested salts as well as applied concentration. The results show that salts with lithocholic acid **3a**–**g** in concentrations 10–50 µg/mL decreased the number of CRL-1475 cells compared to the control (Figure 6A). However, besides the salt **3c**, the viability of treated cells was around 70–80%, which suggests good compatibility of the tested agent. The highest reduction of fibroblasts viability is observed for compounds **3c** and **3h** in the range of used concentrations (the lowest IC50 values). Interestingly, the noncytotoxic effect is noticed for salts that have 12 or 16 carbon atoms in the alkyl chain (**3i**–**j**), which are probably associated with the chemical structure and physicochemical nature of the tested agents.

In the case of the **2** derivative salts, the viability of CRL-1475 cells was found around 75–80% for **4a**–**d**, **4f**, and **4h**–**j** compounds in the range of used concentrations (Figure 6B). The application of **4e** and **4g** salts cause a decrease in the viability of tested cells to 50–55% when applied at the highest concentrations (the lowest IC50 values). Moreover, compounds **4h**–**j** with 8, 12 or 16 carbon atoms caused the weakest cytotoxic effect on tested cells. This indicates that increasing the number of carbon atoms may result in weaker permeability into fibroblast cells. The comparison of IC50 values for two types of tested salts showed that salts based on lithocholic acid showed a slightly stronger cytotoxic effect against CRL-1475 cells compared to the salts based on steroid **2** (Table 6). 

As indicated in a paper published by López-García et al. in accordance with ISO 10993-5, percentages of cell viability above 80% are considered to have non-cytotoxicity; within 80–60%, weak; 60–40%, moderate; and below 40%, strong cytotoxicity, respectively [42]. In effect, it could be interpreted that, in the majority of cases, the percentages of viable cells were high and, consequently, these agents were harmless or exerted weak toxicity.

## 3. Materials and Methods

### 3.1. General Remarks

^1^H and ^13^C NMR spectra were recorded on a Bruker Avance II spectrometer (400 and 100 MHz, respectively). Melting points were determined on an MP70 (Mettler-Toledo GmbH, Greifensee, Switzerland) apparatus and were uncorrected. Mass spectra were obtained with an Accurate-Mass Q-TOF LC/MS 6530 spectrometer. IR spectra were recorded on a Nicolet series II Magna-IR 550 FT-IR spectrometer (Thermo Scientific, Waltham, MA, USA). Steroidal compounds 3α-hydroxy-24-(*N*-imidazolyl)-5β-cholane (**5**), 22-(*N*-imidazolyl)-23,24-dinorchol-4-en-3-one (**6**), and imidazolium salts **3a**, **3b**, **4a**, **4b** were prepared according to literature procedure [34]. Other chemicals are commercially available and were used as received. Copies of spectra of new compounds are included in the Supplementary Materials.

### 3.2. General Procedure for the Synthesis of Imidazolium Salts

Alkyl iodide (excess) was added to *N*-imidazolyl steroid (**5** or **6**) under argon. The reaction was carried out at room or elevated temperature. After complete conversion, the product was precipitated with Et_2_O (10 mL) and filtered. The excess of alkyl iodide was then washed by n-pentane. The remaining residue was dissolved in CH_2_Cl_2_ (2 mL) and the product was precipitated with Et_2_O (10 mL) and filtered. The crystallization (CH_2_Cl_2_/Et_2_O) was repeated two more times.

#### 3.2.1. *N*-(3α-Hydroxy-5β-cholan-24-yl)-*N*′-propylimidazolium Iodide (**3c**)

General procedure was followed using **5** (50 mg, 0.12 mmol) and propyl iodide (0.5 mL) at 80 °C to produce, after 2 h, a white salt in 74% yield (52 mg). Mp = 175.5–176.3 °C; IR (ATR) ν = 3345, 2931, 2864, 1555, 1443, 1371, 1156, 1030 cm^−1^; ^1^H NMR (400 MHz, CDCl_3_) δ 10.19 (s, 1H), 7.50 (s, 1H), 7.43 (s, 1H), 4.29 (s, 2H), 4.27 (s, 2H), 3.62 (m, 1H), 0.91 (s, 3H), 0.62 (s, 3H) ppm; ^13^C NMR (100 MHz, CDCl_3_) δ 137.2, 121.7, 121.5, 71.8, 56.4, 55.8, 51.7, 50.8, 42.7, 42.1, 40.4, 40.1, 36.4, 35.8, 35.3, 34.5, 32.3, 30.5, 29.7, 28.4, 27.2, 27.0, 26.4, 24.2, 23.7, 23.3, 20.8, 18.6, 12.1, 10.8 ppm; ESI-HRMS *m*/*z*: calcd for [M−I]^+^ C_30_H_51_N_2_O^+^ 455.3996, found 455.4011.

#### 3.2.2. *N*-Butyl-*N*′-(3α-hydroxy-5β-cholan-24-yl)imidazolium Iodide (**3d**)

General procedure was followed using **5** (50 mg, 0.12 mmol) and butyl iodide (0.5 mL) at 80 °C to produce, after 2 h, a white salt in 71% yield (51 mg). Mp = 178.1–179.4 °C; IR (ATR) ν = 3349, 2927, 2861, 1556, 1444, 1371, 1155, 1031 cm^−1^; ^1^H NMR (400 MHz, CDCl_3_) δ 10.05 (s, 1H), 7.55 (s, 1H), 7.49 (s, 1H), 4.34 (m, 2H), 4.28 (m, 2H), 3.58 (m, 1H), 0.86 (s, 3H), 0.58 (s, 3H) ppm; ^13^C NMR (100 MHz, CDCl_3_) δ 136.1, 122.3, 122.1, 71.4, 56.3, 55.7, 50.4, 49.7, 42.5, 41.8, 40.2, 40.0, 36.2, 35.6, 35.2, 34.4, 32.1, 32.0, 30.4, 29.7, 28.2, 27.0, 26.9, 26.2, 24.0, 23.2, 20.6, 19.3, 18.5, 13.4, 11.9 ppm; ESI-HRMS *m*/*z*: calcd for [M−I]^+^ C_31_H_53_N_2_O^+^ 469.4152, found 469.4167.

#### 3.2.3. *N*-(3α-Hydroxy-5β-cholan-24-yl)-*N*′-pentylimidazolium Iodide (**3e**)

General procedure was followed using **5** (50 mg, 0.12 mmol) and pentyl iodide (0.5 mL) at 80 °C to produce, after 2 h, a white salt in 72% yield (53 mg). The spectral data are consistent with those given in the literature [34].

#### 3.2.4. *N*-Hexyl-*N*′-(3α-hydroxy-5β-cholan-24-yl)imidazolium Iodide (**3f**)

General procedure was followed using **5** (50 mg, 0.12 mmol) and hexyl iodide (0.5 mL) at 80 °C to produce, after 2 h, a white salt in 72% yield (54 mg). The spectral data are consistent with those given in the literature [34].

#### 3.2.5. *N*-Heptyl-*N*′-(3α-hydroxy-5β-cholan-24-yl)imidazolium Iodide (**3g**)

General procedure was followed using **5** (50 mg, 0.12 mmol) and heptyl iodide (0.5 mL) at 80 °C to produce, after 2 h, a white salt in 70% yield (54 mg). Mp = 183.5–184.2 °C; IR (ATR) ν = 3351, 2929, 2858, 1555, 1441, 1373, 1155, 1032 cm^−1^; ^1^H NMR (400 MHz, CDCl_3_) δ 10.09 (s, 1H), 7.50 (s, 1H), 7.49 (s, 1H), 4.33 (m, 4H), 3.59 (m, 1H), 0.87 (s, 3H), 0.59 (s, 3H) ppm; ^13^C NMR (100 MHz, CDCl_3_) δ 136.2, 122.2, 122.1, 71.5, 56.3, 55.7, 50.4, 50.1, 42.6, 41.9, 40.2, 40.0, 36.3, 35.7, 34.4, 32.1, 31.4, 30.4, 30.2, 29.7, 29.6, 28.5, 28.3, 27.03, 27.0, 26.3, 26.0, 24.0, 23.2, 22.4, 20.7, 18.5, 13.9, 11.9 ppm; ESI-HRMS *m*/*z*: calcd for [M−I]^+^ C_34_H_59_N_2_O^+^ 511.4622, found 511.4642.

#### 3.2.6. *N*-(3α-Hydroxy-5β-cholan-24-yl)-*N*′-octylimidazolium Iodide (**3h**)

General procedure was followed using **5** (50 mg, 0.12 mmol) and octyl iodide (0.5 mL) at 80 °C to produce, after 2 h, a white salt in 74% yield (58 mg). Mp = 185.5–185.9 °C; IR (ATR) ν = 3352, 2930, 2859, 1553, 1442, 1374, 1152, 1030 cm^−1^; ^1^H NMR (400 MHz, CDCl_3_) δ 10.23 (s, 1H), 7.37 (s, 1H), 7.35 (s, 1H), 4.36 (m, 2H), 4.33 (m, 2H), 3.62 (m, 1H), 0.91 (s, 3H), 0.63 (s, 3H) ppm; ^13^C NMR (100 MHz, CDCl_3_) δ 136.3, 122.2, 121.9, 71.4, 56.5, 55.7, 50.3, 50.1, 42.6, 42.0, 40.3, 40.0, 36.3, 35.8, 34.3, 32.1, 31.6, 30.4, 30.2, 29.7, 29.6, 29.3, 28.6, 28.3, 27.1, 27.0, 26.4, 26.0, 24.2, 23.3, 22.4, 20.5, 18.4, 13.8, 11.7 ppm; ESI-HRMS *m*/*z*: calcd for [M−I]^+^ C_35_H_61_N_2_O^+^ 525.4778, found 525.4802.

#### 3.2.7. *N*-Dodecyl-*N*′-(3α-hydroxy-5β-cholan-24-yl)imidazolium Iodide (**3i**)

General procedure was followed using **5** (50 mg, 0.12 mmol) and dodecyl iodide (0.5 mL) at 80 °C to produce, after 2 h, a white salt in 64% yield (54 mg). Mp = 121.2–123.5 °C; IR (ATR) ν = 3348, 2925, 2858, 1556, 1444, 1371, 1155, 1031 cm^−1^; ^1^H NMR (400 MHz, CDCl_3_) δ 10.09 (s, 1H), 7.47 (s, 1H), 7.45 (s, 1H), 4.31 (m, 4H), 3.62 (m, 1H), 0.87 (s, 3H), 0.59 (s, 3H) ppm; ^13^C NMR (100 MHz, CDCl_3_) δ 136.5, 122.1, 121.6, 71.3, 56.7, 55.6, 50.5, 50.3, 42.6, 42.0, 40.4, 40.1, 36.5, 35.9, 35.4, 34.5, 32.3, 31.8, 30.5, 30.4, 30.2, 29.7, 29.6, 29.5, 29.4, 29.3, 29.0, 28.1, 27.2, 27.0, 26.5, 26.3, 24.3, 23.5, 22.4, 20.4, 18.6, 13.9, 12.1 ppm; ESI-HRMS *m*/*z*: calcd for [M−I]^+^ C_39_H_69_N_2_O^+^ 581.5404, found 581.5429.

#### 3.2.8. *N*-Hexadecyl-*N*′-(3α-hydroxy-5β-cholan-24-yl)imidazolium Iodide (**3j**)

General procedure was followed using **5** (50 mg, 0.12 mmol) and hexadecyl iodide (0.5 mL) at 80 °C to produce, after 2 h, a white salt in 55% yield (50 mg). Mp = 185.5–186.7 °C; IR (ATR) ν = 3351, 2932, 2861, 1556, 1443, 1371, 1152, 1031 cm^−1^; ^1^H NMR (400 MHz, CDCl_3_) δ 10.27 (s, 1H), 7.35 (s, 1H), 7.33 (s, 1H), 4.36 (m, 4H), 3.63 (m, 1H), 0.91 (s, 3H), 0.63 (s, 3H) ppm; ^13^C NMR (100 MHz, CDCl_3_) δ 136.8, 121.8, 121.7, 71.7, 56.4, 55.8, 50.7, 50.3, 42.7, 42.0, 40.4, 40.1, 36.4, 35.8, 35.3, 34.5, 32.3, 31.9, 30.5, 30.4, 30.3, 30.25, 30.2, 29.7, 29.6, 29.59, 29.5, 29.4, 29.3, 29.2, 29.0, 28.3, 27.1, 27.0, 26.4, 26.2, 24.1, 23.3, 22.6, 20.8, 18.6, 14.1, 12.0 ppm; ESI-HRMS *m*/*z*: calcd for [M−I]^+^ C_43_H_77_N_2_O^+^ 637.6030, found 637.6051.

#### 3.2.9. *N*-(3-Oxo-23,24-dinorchol-4-en-22-yl)-*N*′-propylimidazolium Iodide (**4c**)

General procedure was followed using **6** (50 mg, 0.13 mmol), propyl iodide (1 mL) at 80 °C to produce, after 15 min, a white salt in quantitative yield (74 mg). Mp = 198.5–201.5 °C; IR (ATR) ν = 2933, 2873, 1655, 1560, 1444, 1348, 1167 cm^−1^; ^1^H NMR (400 MHz, CDCl_3_) *δ* 10.06 (s, 1H), 7.60 (s, 1H), 7.43 (s, 1H), 5.63 (s, 1H), 4.30 (m, 3H), 3.98 (dd, *J* = 13.4, 9.9 Hz, 1H), 1.11 (s, 3H),0.92 (t, *J* = 7.4 Hz, 3H), 0.85 (d, *J =* 6.5 Hz, 3H), 0.71 (s, 3H) ppm; ^13^C NMR (100 MHz, CDCl_3_) *δ* 199.3, 171.2, 136.4, 123.5, 122.6, 122.4, 55.2, 55.2, 53.3, 53.2, 51.2, 42.6, 39.1, 38.3, 37.3, 35.4, 35.2, 33.7, 32.6, 31.6, 27.8, 24.0, 23.5, 20.7, 17.1, 16.3, 12.0, 10.5 ppm; ESI-HRMS *m/z*: calcd for [M−I]^+^ 423.3370, found 423.2445.

#### 3.2.10. *N*-Butyl-*N*′-(3-oxo-23,24-dinorchol-4-en-22-yl)imidazolium Iodide (**4d**)

General procedure was followed using **6** (50 mg, 0.13 mmol), butyl iodide (1 mL, 8.80 mmol) at 80 °C to produce, after 1 h, a white salt in 85% yield (63 mg). Mp = 113.2–120.9 °C; IR (ATR) ν = 3416, 2930, 2852, 1655, 1563, 1454, 1355, 1164 cm^−1^; ^1^H NMR (400 MHz, CDCl_3_) *δ* 10.11 (s, 1H), 7.57 (s, 1H), 7.43 (s, 1H), 5.66 (s, 1H), 4.35 (m, 3H), 3.99 (dd, J = 13.5, 9.9 Hz, 1H), 1.13 (s, 3H),0.92 (t, *J* = 7.4 Hz, 3H), 0.87 (d, *J =* 6.5 Hz, 3H), 0.73 (s, 3H, CH_3_) ppm; ^13^C NMR (100 MHz, CDCl_3_) *δ* 199.3, 171.2, 136.6, 123.6, 122.6, 122.3, 55.3, 53.4, 53.3, 53.2, 49.7, 42.7, 39.1, 38.3, 37.4, 35.5, 35.3, 33.8, 32.6, 32.0, 31.7, 27.9, 24.0, 20.7, 19.2, 17.2, 16.4, 13.3, 12.1 ppm; ESI-HRMS *m/z*: calcd for [M−I]^+^ 437.3526, found 437.3505.

#### 3.2.11. *N*-(3-Oxo-23,24-dinorchol-4-en-22-yl)-*N*′-pentylimidazolium Iodide (**4e**)

The general procedure was followed using **6** (50 mg, 0.13 mmol), pentyl iodide (1 mL, 7.68 mmol) at 80 °C to produce, after 1.5 h, a white salt with quantitative yield (46 mg). The spectroscopic data are consistent with the literature [34].

#### 3.2.12. *N*-Hexyl-*N*′-(3-oxo-23,24-dinorchol-4-en-22-yl)imidazolium Iodide (**4f**)

The general procedure was followed using **6** (50 mg, 0.13 mmol), hexyl iodide (1 mL, 6.79 mmol) at 80 °C to produce, after 1 h, a white salt in quantitative yield (60 mg). The spectroscopic data are consistent with the literature [34].

#### 3.2.13. *N*-Heptyl-*N*′-(3-oxo-23,24-dinorchol-4-en-22-yl)imidazolium Iodide (**4g**)

General procedure was followed using **6** (83 mg, 0.22 mmol), 1-iodoheptane (1 mL) at 100 °C to produce, after 2 h, a white salt in 73% yield (97 mg). Mp = 100.8 °C (with decomposition); IR (ATR) ν = 3457, 2929, 2852, 1660, 1561, 1449, 1354, 1162 cm^−1^; ^1^H NMR (400 MHz, CDCl_3_) *δ* 10.04 (s, 1H), 7.55 (s, 1H), 7.45 (s, 1H), 5.62 (s, 1H), 4.30 (m, 3H), 3.97 (dd, J = 12.9, 9.9 Hz, 1H), 1.09 (s, 3H), 0.83 (d, *J =* 6.4 Hz, 3H), 0.77 (m, 3H), 0.70 (s, 3H) ppm; ^13^C NMR (100 MHz, CDCl_3_) *δ* 199.2, 171.2, 136.3, 123.4, 122.6, 122.2, 55.1, 55.04, 53.2, 53.1, 49.8, 42.5, 39.0, 38.2, 37.2, 35.3, 35.2, 33.6, 32.5, 31.5, 31.2, 30.0, 28.3, 27.7, 25.8, 23.9, 22.1, 20.6, 17.0, 16.2, 13.7, 11.9 ppm; ESI-HRMS *m/z*: calcd for [M−I]^+^ 479.3996, found 479.3957.

#### 3.2.14. *N*-Octyl-*N*′-(3-oxo-23,24-dinorchol-4-en-22-yl)imidazolium Iodide (**4h**)

General procedure was followed using **6** (75 mg, 0.20 mmol), 1-iodooctane (1 mL) at 100 °C to produce, after 2 h, a white salt in 93% yield (114 mg). Mp = 101.6 °C; IR (ATR) ν = 3455, 2925, 2851, 1660, 1560, 1449, 1354, 1162 cm^−1^; ^1^H NMR (400 MHz, CDCl_3_) *δ* 10.10 (s, 1H), 7.55 (s, 1H), 7.45 (s, 1H), 5.64 (s, 1H), 4.32 (m, 3H), 3.99 (dd, *J =* 13.4, 10.0 Hz, 1H), 1.17 (s, 3H), 1.11 (s, 3H), 0.86 (d, *J* = 5.7 Hz, 3H), 0.72 (s, 3H) ppm; ^13^C NMR (100 MHz, CDCl_3_) *δ* 199.3, 171.2, 136.4, 123.5, 122.6, 122.2, 55.2, 55.2, 53.3, 53.2, 49.9, 42.6, 39.1, 38.3, 37.3, 35.4, 35.2, 33.7, 32.6, 31.6, 31.4, 30.1, 28.8, 28.7, 27.8, 25.9, 24.0, 22.3, 20.7, 17.1, 16.4, 13.8, 12.1 ppm; ESI-HRMS *m/z*: calcd for [M−I]^+^ 493.4152, found 493.4128.

#### 3.2.15. *N*-Dodecyl-*N*′-(3-oxo-23,24-dinorchol-4-en-22-yl)imidazolium Iodide (**4i**)

General procedure was followed using **6** (80 mg, 0.21 mmol), 1-iodododecane (1 mL) at 100 °C to produce, after 2 h, a white salt in 81% yield (115 mg). Mp = 177.4–180.4 °C; IR (ATR) ν = 3454, 2920, 2850, 1673, 1561, 1350, 1455, 1169 cm^−1^; ^1^H NMR (400 MHz, CDCl_3_) *δ* 10.11 (s, 1H), 7.53 (s, 1H), 7.44 (s, 1H), 5.66 (s, 1H), 4.33 (m, 3H), 3.99 (m, 1H), 1.19 (s, 3H), 0.87 (d, *J* = 6.5 Hz, 3H), 0.81 (t, *J* = 6.8 Hz, 3H), 0.73 (s, 3H) ppm; ^13^C NMR (100 MHz, CDCl_3_) *δ* 199.3, 171.2, 136.6, 123.6 (2C), 122.6, 122.2, 55.3, 55.2, 53.4, 53.2, 50.0, 42.7 (2C), 39.1, 38.3, 37.4, 35.4, 35.3, 33.7, 32.6, 31.7, 30.1, 29.4, 29.3, 29.2, 29.1, 28.8, 27.9, 26.0, 24.0, 22.5, 20.7, 17.1, 16.4, 13.9, 12.0 ppm; ESI-HRMS *m/z*: calcd for [M−I]^+^ 549.4778, found 549.4784.

#### 3.2.16. *N*-Hexadecyl-*N*′-(3-oxo-23,24-dinorchol-4-en-22-yl)imidazolium Iodide (**4j**)

General procedure was followed using **6** (80 mg, 0.21 mmol), 1-iodohexadecane (1 mL) and CH_2_Cl_2_ (1 mL) at 100 °C to produce, after 2 h, a white salt in 67% yield (103 mg). Mp = 175.6–180.3 °C; IR (ATR) ν = 3473, 2920, 2850, 1665, 1561, 1455, 1353, 1164 cm^−1^; ^1^H NMR (400 MHz, CDCl_3_) *δ* 10.17 (s, 1H), 7.51 (s, 1H), 7.42 (s, 1H), 5.68 (s, 1H), 4.34 (m, 3H), 4.00 (dd, *J =* 13.5, 9.8 Hz, 1H), 1.20 (s, 3H), 0.88 (d, *J* = 6.5 Hz, 3H), 0.84 (m, 3H), 0.75 (s, 3H) ppm; ^13^C NMR (100 MHz, CDCl_3_) *δ* 199.3, 171.1, 136.6, 123.6 (2C), 122.6, 122.2, 55.3, 55.2, 53.4, 53.2, 50.0, 42.7 (2xC), 39.2, 38.3, 37.4, 35.5, 35.3, 33.8, 32.6, 31.7, 30.1, 29.5, 29.5, 29.5, 29.4, 29.3, 29.2, 29.2, 28.8, 27.9, 26.0, 24.0, 22.5, 20.8, 17.2, 16.4, 13.9, 12.1 ppm; HR-MS (ESI+) *m/z*: calcd for [M−I]^+^ 605.5404, found 605.5356.

### 3.3. Antimicrobial Activity

The antibacterial and antifungal activity of imidazolium salts were evaluated by broth microdilution assay in 96-well plates. The two-fold serial microdilution assay, described by the Clinical and Laboratory Standards Institute, was performed for the measurements of the minimal inhibitory concentrations (MICs) expressed in μg/mL [43,44]. The imidazolium salt was first dissolved in dimethyl sulfoxide (DMSO) and incorporated into Mueller-Hinton broth (MHB) for bacteria and into Roswell Park Memorial Institute 1640 medium (RPMI 1640) for fungi to obtain a concentration of 1024 μg/mL, with the final solution composition being 95% MHB or RPMI 1640 and 5% DMSO by volume. The salts were then serially two-fold diluted to obtain concentrations ranging from 512 to 0.06 μg/mL in wells containing MHB where the bacteria were tested, or in wells containing RPMI 1640 where fungi were tested. Then, each diluted sample (50 μL) was mixed with 50 μL of inoculums of the tested microorganisms to achieve an initial inoculum of approximately 106 CFU/mL and incubated at 35 °C for 24 h for bacteria, and 25 °C for 48–72 h for fungi. The experiments were performed in duplicate. The MIC value was determined as the lowest concentration of the salt that inhibits visible growth after incubation. The following reference strains were tested: *Staphylococcus aureus* ATCC 25923, *Bacillus cereus* ATCC 11778, *Escherichia coli* DSM 10233, *Candida albicans* ATCC 10231, *Aspergillus niger* ATCC 16888, *Aspergillus fumigatus* ATCC 204305, *Trichophyton mentagrophytes* ATCC 9533, *Cryptococcus neoformans* ATCC 14116, and *Microsporum canis* ATCC 11621.

### 3.4. Hemocompatibility Studies

To evaluate the ability of the tested agents to release the hemoglobin from treated cells, fresh human red blood cells (RBCs) were obtained from healthy volunteers. For this purpose, the collected cells were suspended in phosphate-buffered saline (PBS) to establish hematocrit ~5%. The tested salts were added at a concentration range of 5–50 µg/mL and incubated for 1 h at 37 °C. After centrifugation, the relative hemoglobin concentration in the supernatants was spectrophotometrically assessed at a wavelength of 540 nm. The 0% hemolysis was taken from samples after the addition of 10 μL PBS, while the 100% hemolysis was taken from samples in which 1% Triton X-100 was added to disrupt all cell membranes. The HC50 values were calculated using nonlinear regression in GraphPad Prism.

### 3.5. Cytotoxicity Studies

Human monocytic cell line THP-1 cells (ATCC, TIB-202) was cultured in Roswell Park Memorial Institute medium (RPMI-1640, ATCC) supplemented with 10% heat-inactivated fetal bovine serum (Eurx, Gdańsk, Poland), 1% penicillin/streptomycin mixture (Gibco, Thermo Fisher Scientific, Inc., Waltham, MA, USA), and 2-mercaptoethanol (0.05 mM) (Gibco, Thermo Fisher Scientific, Inc., Waltham, MA, USA).

To examine the metabolic activity of THP-1 cells after treatment by tested salts, the resazurin assay was used. The cells were incubated with synthesized agents at a concentration of 5–100 µg/mL, 24 h exposure; 10 µL of reagent was added to each well. The cells were then incubated for 3 h in the dark at 37 °C with a 5% CO_2_ atmosphere. The absorbance was measured at 570 nm using a microplates reader (Varioscan lux Thermofisher) and calculated as a percentage of control.

The cytotoxicity of imidazolium salts was evaluated against fibroblast CRL-1475 cell lines originating from American Type Culture Collection (Manassas, VA, USA). Cells were grown in 96-well plates at 5–7 × 103 cells per well to full confluence in RPMI-1640 medium with 10% fetal bovine serum (FBS), 50 U/mL penicillin, 50 mg/mL streptomycin under physiological conditions, at 37 °C with 5% CO_2_.

The cytotoxicity of the tested salts was determined by MTT ((3-(4,5-dimethylthiazol-2-yl)-2,5-diphenyltetrazolium bromide) assay method. All salts were dissolved in PBS and diluted in fresh medium to the desired concentrations (10, 30, and 50 µg/mL). After 24 h of cells incubation with tested salts, the MTT assay protocol was followed. The culturing medium was discarded, and the cells were rinsed with PBS three times. The MTT reagent (5 mg/mL) was then added to each well and incubated for 0.5–1 h, depending on the cell line. The medium was removed from the wells and 100 µL of DMSO with 10 µL of Sorren’s buffer (0.1 mol/L glycine with 0.1 mol/L NaCl equilibrated to pH 10.5) was added. The optical density was recorded with a BioTek Epoch plate reader spectrophotometer at a wavelength of 570 nm. Values are described as a percent of control.

The IC50 values for treated cells were calculated using nonlinear regression in GraphPad Prism.

### 3.6. Statistical Analysis

Statistical analysis was performed using Statistica v. 13.3 (StatSoft, Tulsa, OK, USA). Intergroup statistical comparisons were analyzed using a one-way analysis of variance (ANOVA). Differences were considered significant when *p* < 0.05.

## 4. Conclusions

Two series of imidazolium salts based on lithocholic acid and 3-oxo-23,24-dinorchol-4-en-22-al derivatives were synthesized as a supplement to the previously reported compounds. Optimization of the conditions allowed the efficient synthesis of known salts and the additional synthesis of twelve new salts. The new compounds (20 in total) were tested against human life-threatening pathogens, especially fungal species as well as bacterial pathogens, which are frequently co-existing during fungal infections, and vice versa. Our results suggest that synthesized salts possess a wide range of antimicrobial activity, which makes them very attractive candidates for further study, including for the co-infection model.

The results from hemolytic activity indicate low hemolysis at a concentration corresponding to MIC value. It was also found that the synthesized salts do not affect the metabolic activity representative of the immune cells. Moreover, it should be emphasized that incubation of synthesized agents with fibroblast cells caused 20% reduction of the survival, which classified them as innoxious substances. In effect, it could be concluded that the tested salts show relative compatibility with host cells.

In summary, steroid-based imidazolium salts showed a broad spectrum of activity against human pathogens. Taking into account that the MIC values were much lower in most of the tested compounds than hemolytic activity, the collected results are very promising. It should be noted that salts with propyl and butyl substituents were much more active against *C. albicans* than Amphotericin B, which is a very toxic antifungal drug [45].

## Figures and Tables

**Table 1:** Synthesis of steroid-based salts.

Salt	Reaction Temperature	Reaction Time	Yield	Salt	Reaction Temperature	Reaction Time	Yield	Number of Carbon Atoms in the Alkyl Substituent
**3a ^a^**	r.t.	24 h	83%	**4a ^a^**	r.t.	20 h	100%	1
**3b ^a^**	r.t.	24 h	64%	**4b ^a^**	r.t.	20 h	99%	2
**3c**	80 °C	2 h	74%	**4c**	80 °C	0.25 h	99%	3
**3d**	80 °C	2 h	71%	**4d**	80 °C	1 h	85%	4
**3e**	80 °C	2 h	72%	**4e**	80 °C	1 h	79%	5
**3f**	80 °C	2 h	72%	**4f**	80 °C	1.5 h	100%	6
**3g**	80 °C	2 h	70%	**4g**	80 °C	3 h	73%	7
**3h**	80 °C	2 h	74%	**4h**	100 °C	2 h	93%	8
**3i**	80 °C	2 h	64%	**4i**	100 °C	1.5 h	81%	12
**3j**	80 °C	2 h	55%	**4j**	100 °C	2 h	67%	16

^a^ recently published results [34].

**Table 2:** Minimum inhibitory concentration (MIC) values of imidazolium salts against bacterial species.

MIC (µg/mL)							
Salt	*S. aureus*	*B. cereus*	*E. coli*	Salt	*S. aureus*	*B. cereus*	*E. coli*
**3a**	4 ^a^	32 ^a^	16 ^a^	**4a**	32 ^a^	64 ^a^	16 ^a^
**3b**	2 ^a^	16 ^a^	16 ^a^	**4b**	16 ^a^	32 ^a^	8 ^a^
**3c**	1	8	16	**4c**	16	16	8
**3d**	0.5	4	16	**4d**	8	16	8
**3e**	1 ^a^	4 ^a^	16 ^a^	**4e**	4 ^a^	4 ^a^	4 ^a^
**3f**	0.5 ^a^	2 ^a^	16 ^a^	**4f**	2 ^a^	2 ^a^	4 ^a^
**3g**	≤0.06	2	16	**4g**	0.5	0.5	4
**3h**	≤0.06	2	64	**4h**	0.25	0.5	4
**3i**	2	4	≥512	**4i**	0.5	1	≥512
**3j**	16	64	≥512	**4j**	4	8	≥512
Ampicillin	0.25–1	0.25–0.5	2–8				

^a^ recently published results [34].

**Table 3:** Minimum inhibitory concentration (MIC) values of imidazolium salts against fungal species.

MIC (µg/mL)						
Salt	*C. albicans*	*A. niger*	*A. fumigatus*	*T. mentagrophytes*	*C. neoformans*	*M. canis*
**3a**	0.25 ^a^	4	4	0.25	1	0.25
**3b**	0.5 ^a^	1	1	0.5	1	0.25
**3c**	0.25	16	8	2	2	0.25
**3d**	0.5	16	8	2	4	0.25
**3e**	0.5 ^a^	2	8	0.5	2	0.25
**3f**	0.5 ^a^	4	8	0.5	4	0.25
**3g**	0.5	8	16	1	8	0.5
**3h**	1	8	16	2	8	1
**3i**	8	64	16	4	32	2
**3j**	32	64	64	32	64	16
**4a**	2 ^a^	8	2	2	4	0.5
**4b**	1 ^a^	2	4	2	2	0.25
**4c**	≤0.06	16	8	2	2	0.5
**4d**	≤0.06	8	8	1	2	0.25
**4e**	2 ^a^	8	8	2	4	0.5
**4f**	1 ^a^	2	8	1	2	0.25
**4g**	0.5	8	16	1	4	0.25
**4h**	0.5	4	16	1	4	0.25
**4i**	0.5	4	4	1	4	0.25
**4j**	8	16	32	4	32	2
Fluconazole	0.5	16	64	64	4	32
Amphotericin B	0.5	2	2	1	0.5	1
Voriconazole	0.25	0.5	0.25	0.5	0.125	1

^a^ recently published results [34].

**Table 4:** The calculated logP (ClogP) values of imidazolium salts.

Salt	ClogP	Salt	ClogP
**3a**	3.467	**4a**	1.863
**3b**	3.996	**4b**	2.392
**3c**	4.525	**4c**	2.921
**3d**	5.054	**4d**	3.450
**3e**	5.583	**4e**	3.979
**3f**	6.112	**4f**	4.508
**3g**	7.170	**4g**	5.566
**3h**	8.228	**4h**	6.624
**3i**	9.286	**4i**	7.682
**3j**	11.402	**4j**	9.798

**Table 5:** Hemolytic concentration of 50 (HC50) values of imidazolium salts.

HC50 (µg/mL)			
Salt	RBC	Salt	RBC
**3a**	265.1	**4a**	nd
**3b**	44.6	**4b**	nd
**3c**	54.7	**4c**	nd
**3d**	53.7	**4d**	nd
**3e**	39.9	**4e**	401.4
**3f**	42.3	**4f**	43.9
**3g**	12.2	**4g**	57.6
**3h**	541.6	**4h**	33.8
**3i**	nd	**4i**	54.5
**3j**	nd	**4j**	367.3

nd (not determinable).

**Table 6:** IC50 values and viability at MIC values of imidazolium salts for CRL-1475 and THP-1 cells.

IC50 (µg/mL)						Viability at MIC Value (%)					
Salt	CRL-1475	THP-1	Salt	CRL-1475	THP-1	Salt	CRL-1475	THP-1	Salt	CRL-1475	THP-1
**3a**	107.4	nd	**4a**	469.0	562.9	**3a**	100.6 ± 2.5	111.88 ± 22.5	**4a**	100.6 ± 3.1	112.69 ± 2.59
**3b**	113.9	205.3	**4b**	524.2	nd	**3b**	102.6 ± 1.5	122.4 ± 5.53	**4b**	100.2 ± 2.3	130.51 ± 4.75
**3c**	36.5	452.9	**4c**	562.4	352.1	**3c**	98.0 ± 4.3	95.11 ± 7.09	**4c**	98.2 ± 1.4	105.38 ± 6.42
**3d**	99.2	976.8	**4d**	446.3	96.33	**3d**	101.7 ± 2.3	99.82 ± 16.78	**4d**	101.3 ± 2.0	109.39 ± 4.65
**3e**	100.8	77.43	**4e**	42.9	295.8	**3e**	99.9 ± 2.6	123.59 ± 16.79	**4e**	100.9 ± 1.5	71,49 ± 1.49
**3f**	149.6	nd	**4f**	717.7	313.2	**3f**	102.3 ± 5.4	85.24 ± 16.19	**4f**	99.8 ± 0.6	96.07 ± 13.97
**3g**	134.4	nd	**4g**	51.5	238.4	**3g**	99.7 ± 3.4	85.24 ± 21.53	**4g**	96.2 ± 1.9	87.07 ± 12.86
**3h**	53.6	752.2	**4h**	598.8	336.6	**3h**	102.7 ± 2.9	119.57 ± 15.02	**4h**	96.9 ± 2.2	106.68 ± 5.17
**3i**	631.0	nd	**4i**	457.7	713.9	**3i**	80.5 ± 0.8	120.62 ± 17.80	**4i**	100.2 ± 0.7	101.60 ± 13.22
**3j**	841.1	451.4	**4j**	282.3	853.4	**3j**	101.26 ± 2.7	128.68 ± 14.53	**4j**	81.3 ± 2.4	88.10 ± 19.77

nd (not determinable). The viabilities are presented as the mean values ± SD or SE.

## Data Availability

Not applicable.

## Supplementary Materials

The following are available online at https://www.mdpi.com/article/10.3390/ijms222212180/s1.
