# Supplementary material for: Steroid-Functionalized Imidazolium Salts with an Extended Spectrum of Antifungal and Antibacterial Activity
Source: Int J Mol Sci. 2021 Nov 10;22(22):12180. doi: 10.3390/ijms222212180 (PMC8623970; doi:10.3390/ijms222212180)

SUPPORTING INFORMATION

Steroid-functionalized imidazolium salts with an extended spectrum of antifungal and antibacterial activity

Marta Malinowska[1*], Diana Sawicka[2], Katarzyna Niemirowicz-Laskowska[2], Przemysław Wielgat[3], Halina Car[2], Tomasz Hauschild[4], and Agnieszka Hryniewicka[1,5,*]

[1] *Faculty of Chemistry, University of Bialystok, Ciołkowskiego 1K, 15-245 Białystok, Poland; m.malinowska@uwb.edu.pl (M.M.)*
[2] *Department of Experimental Pharmacology, Medical University of Bialystok, Szpitalna 37, 15-295 Białystok, Poland; diana.sawicka@umb.edu.pl (D.S.), hcar@umb.edu.pl (H.C.), katarzyna.niemirowicz@umb.edu.pl (K.N.-L.)*
[3] *Department of Clinical Pharmacology, Medical University of Bialystok, Waszyngtona 15A, 15-274 Białystok, Poland; przemyslaw.wielgat@umb.edu.pl (PW)*
[4] *Faculty of Biology, University of Bialystok, Ciołkowskiego 1J, 15-245 Białystok, Poland; thausch@uwb.edu.pl (T.H.)*
[5] *Department of Organic Chemistry, Medical University of Bialystok, Mickiewicza 2A, 15-222 Bialystok, Poland; agnieszka.hryniewicka@umb.edu.pl (A.H.)*
* *Correspondence: m.malinowska@uwb.edu.pl, agnieszka.hryniewicka@umb.edu.pl*

Copies of NMR spectra of new compounds

^{1}H NMR spectrum of *N-(3α-hydroxy-5β-cholan-24-yl)-N'-propylimidazolium iodide* (***3c***)………………………………..………….… **S4**

^{13}C NMR spectrum of *N-(3α-hydroxy-5β-cholan-24-yl)-N'-propylimidazolium iodide* (***3c***)…………………………………….………. **S5**

^{1}H NMR spectrum of *N-butyl-N'-(3α-hydroxy-5β-cholan-24-yl)imidazolium iodide* (***3d***)……………………………….………... **S6**

^{13}C NMR spectrum of *N-butyl-N'-(3α-hydroxy-5β-cholan-24-yl)imidazolium iodide* (***3d***)……………………………………….... **S7**

^{1}H NMR spectrum of *N-heptyl-N'-(3α-hydroxy-5β-cholan-24-yl)imidazolium iodide* (***3g***)………………..………………….…….. **S8**

^{13}C NMR spectrum of *N-heptyl-N'-(3α-hydroxy-5β-cholan-24-yl)imidazolium iodide* (***3g***)……………………………...………..…. **S9**

^{1}H NMR spectrum of *N-(3α-hydroxy-5β-cholan-24-yl)-N'-octylimidazolium iodide* (***3h***)…………………………………………… **S10**

^{13}C NMR spectrum of *N-(3α-hydroxy-5β-cholan-24-yl)-N'-octylimidazolium iodide* (***3h***)……………………………………..…… **S11**

^{1}H NMR spectrum of *N-dodecyl-N'-(3α-hydroxy-5β-cholan-24-yl)imidazolium iodide* (***3i***)…………………………………...….… **S12**

^{13}C NMR spectrum of *N-dodecyl-N'-(3α-hydroxy-5β-cholan-24-yl)imidazolium iodide* (***3i***)……………………………….…..….… **S13**

^{1}H NMR spectrum of *N-hexadecyl-N'-(3α-hydroxy-5β-cholan-24-yl)imidazolium iodide* (***3j***)……………………………..….…. **S14**

^{13}C NMR spectrum of *N-hexadecyl-N'-(3α-hydroxy-5β-cholan-24-yl)imidazolium iodide* (***3j***)………………………………….….…. **S15**

^{1}H NMR spectrum of *N-(3-oxo-23,24-dinorchol-4-en-22-yl)-N'-propylimidazolium iodide* (***4c***)…………………………………..… **S16**

^{13}C NMR spectrum of *N-(3-oxo-23,24-dinorchol-4-en-22-yl)-N'-propylimidazolium iodide* (***4c***)…………………………………… **S17**

^{1}H NMR spectrum of *N-butyl-N'-(3-oxo-23,24-dinorchol-4-en-22-yl)imidazolium iodide* (***4d***) ..……………………………….……….. **S18**

^{13}C NMR spectrum of *N-butyl-N'-(3-oxo-23,24-dinorchol-4-en-22-yl)imidazolium iodide* (***4d***) ..……………………………….………. **S19**

^{1}H NMR spectrum of *N-heptyl-N'-(3-oxo-23,24-dinorchol-4-en-22-yl)imidazolium iodide* (***4g***)……………………………………. **S20**

^{13}C NMR spectrum of *N-heptyl-N'-(3-oxo-23,24-dinorchol-4-en-22-yl)imidazolium iodide* (***4g***)…………………………………… **S21**

^{1}H NMR spectrum of *N-octyl-N'-(3-oxo-23,24-dinorchol-4-en-22-yl)imidazolium iodide* (***4h***)………………………………...... **S22**

^{13}C NMR spectrum of *N-octyl-N'-(3-oxo-23,24-dinorchol-4-en-22-yl)imidazolium iodide* (***4h***)……………………………….….. **S23**

^{1}H NMR spectrum of *N-dodecyl-N'-(3-oxo-23,24-dinorchol-4-en-22-yl)imidazolium iodide* (***4i***)…………………………………..….… **S24**
^{13}C NMR spectrum of *N-dodecyl-N'-(3-oxo-23,24-dinorchol-4-en-22-yl)imidazolium iodide* (***4i***)……………………………………….. **S25**
^{1}H NMR spectrum of *N-hexadecyl-N'-(3-oxo-23,24-dinorchol-4-en-22-yl)imidazolium iodide* (***4j***)………………..…………………… **S26**
^{13}C NMR spectrum of *N-hexadecyl-N'-(3-oxo-23,24-dinorchol-4-en-22-yl)imidazolium iodide* (***4j***)……………………………………... **S27**

^{1}H NMR spectrum of *N*-(3α-hydroxy-5β-cholan-24-yl)-*N*’-propylimidazolium iodide (**3c**)

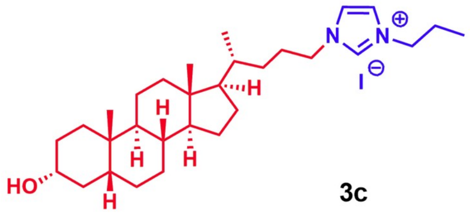

3c

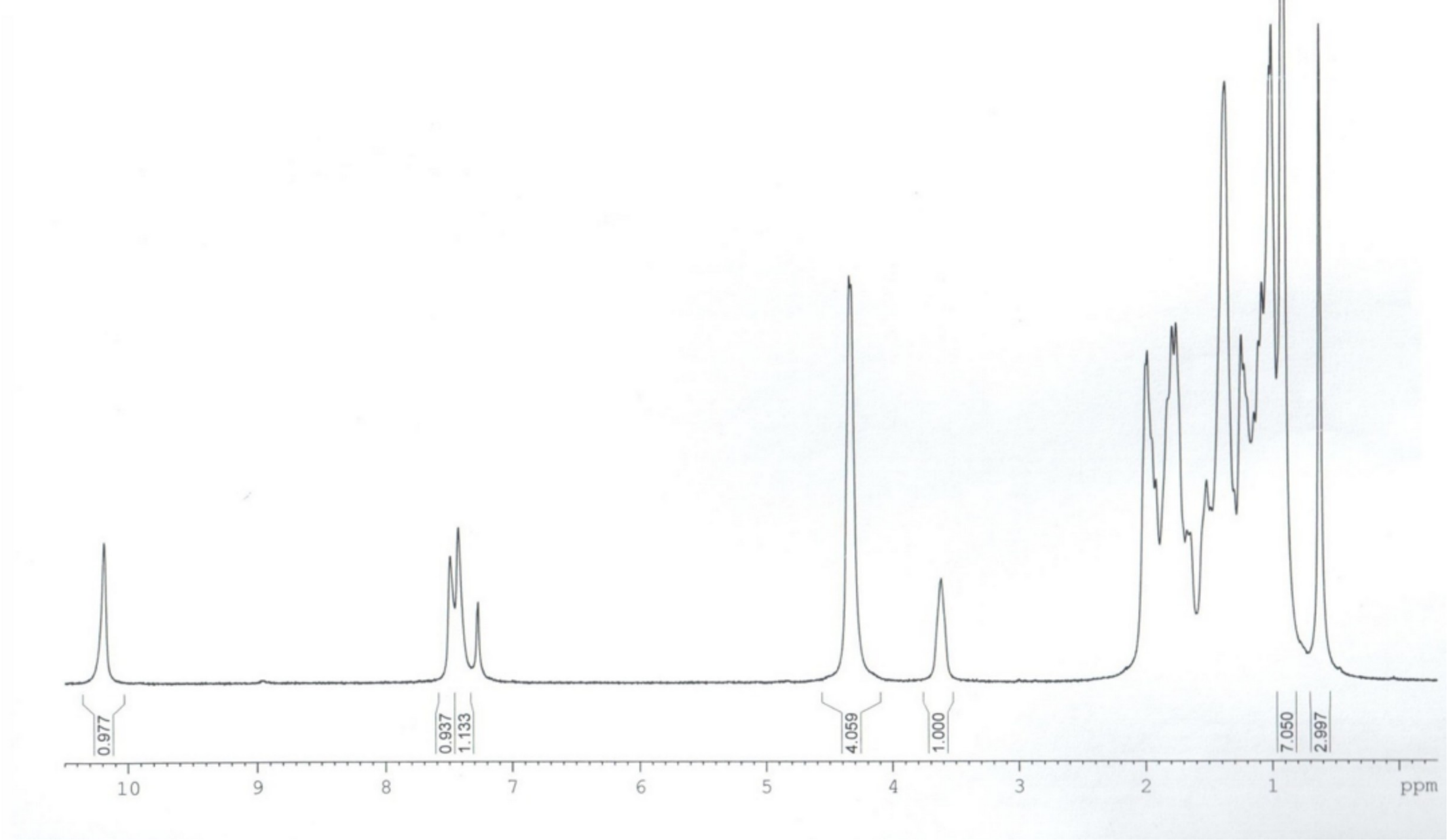

^{13}C NMR spectrum of *N*-(3α-hydroxy-5β-cholan-24-yl)-*N*'-propylimidazolium iodide (**3c**)

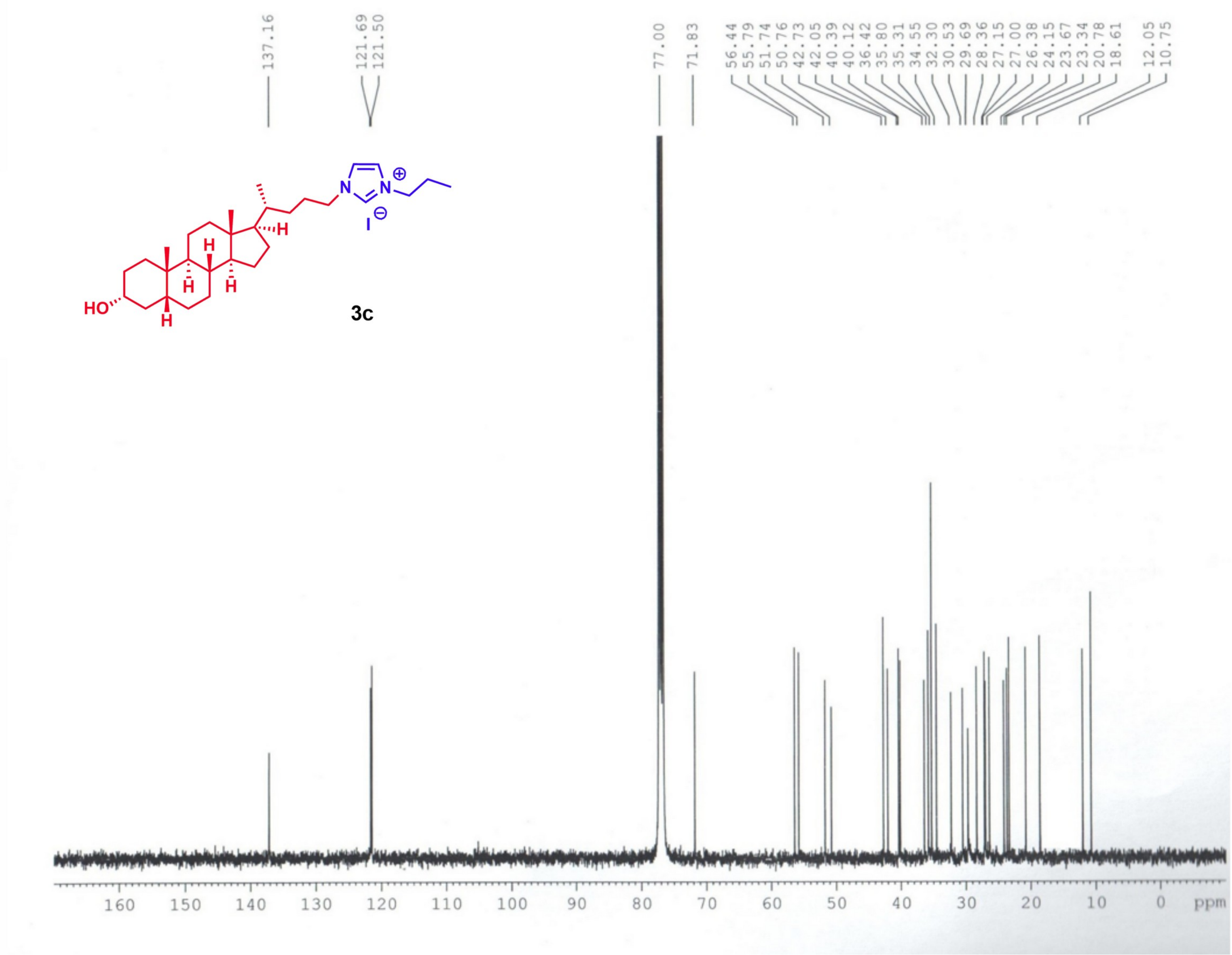

^{1}H NMR spectrum of *N*-butyl-*N*'-(3α-hydroxy-5β-cholan-24-yl)imidazolium iodide (**3d**)

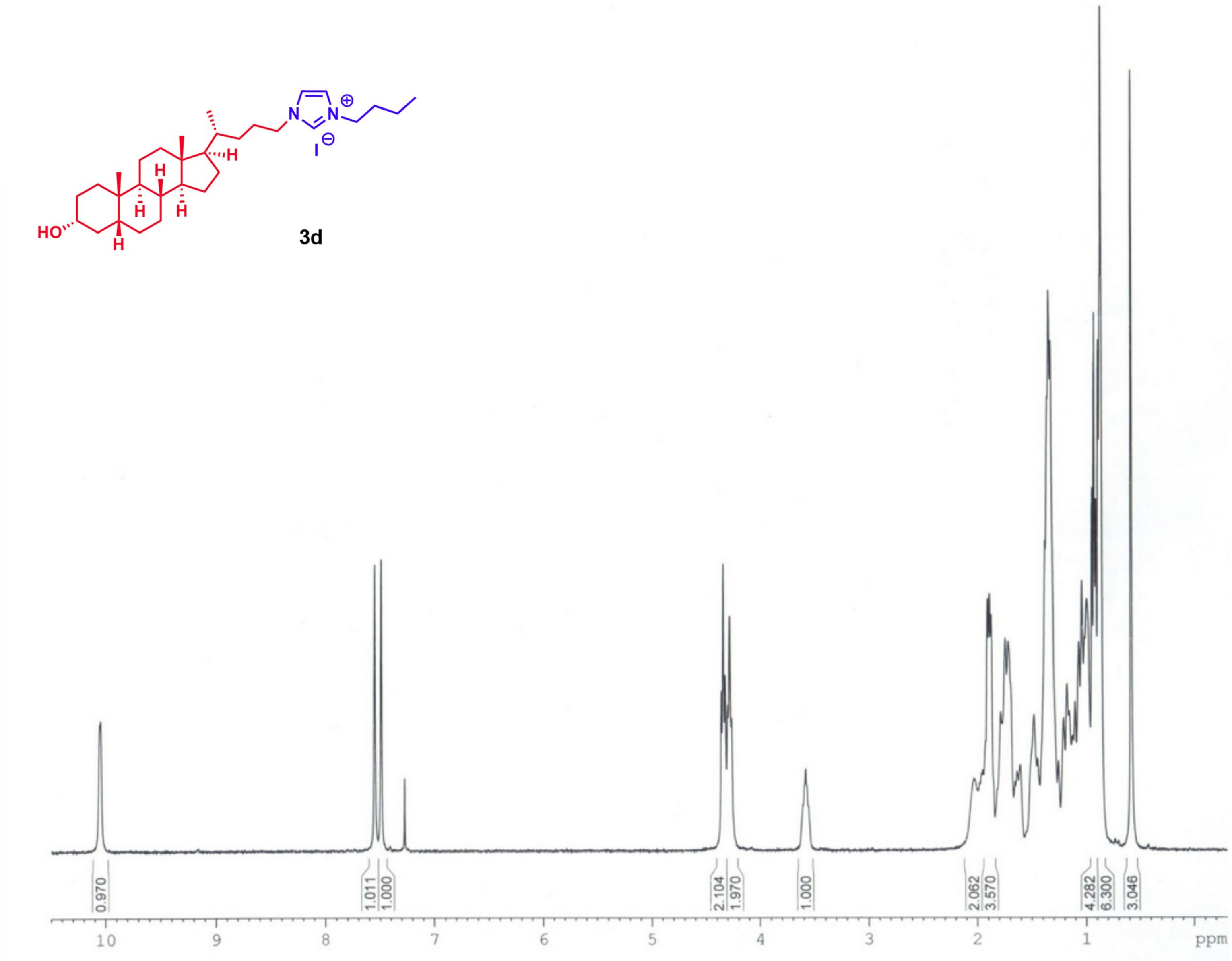

^{13}C NMR spectrum of *N*-butyl-*N'*-(3α-hydroxy-5β-cholan-24-yl)imidazolium iodide (**3d**)

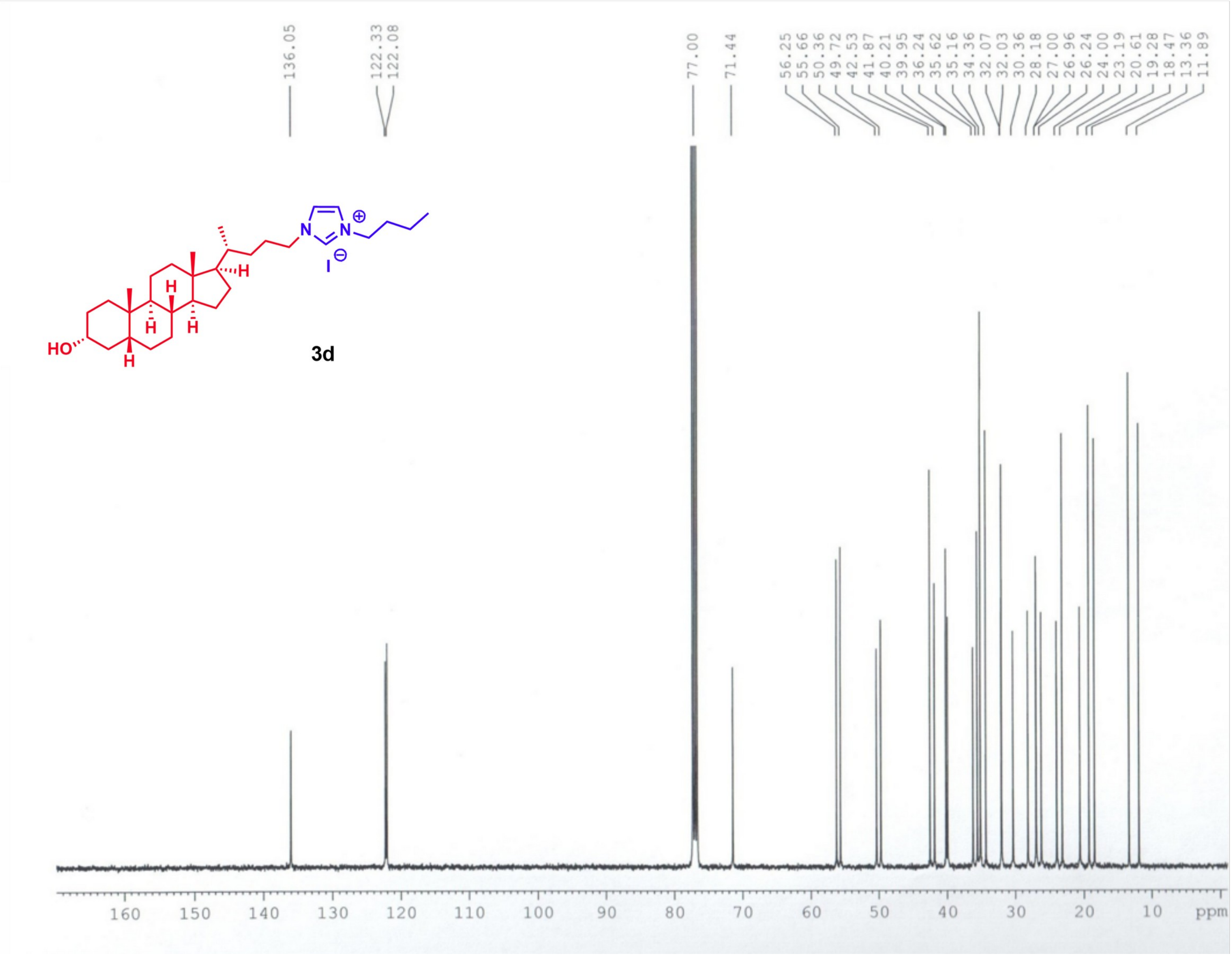

^{1}H NMR spectrum of *N*-heptyl-*N*'-(3α-hydroxy-5β-cholan-24-yl)imidazolium iodide (**3g**)

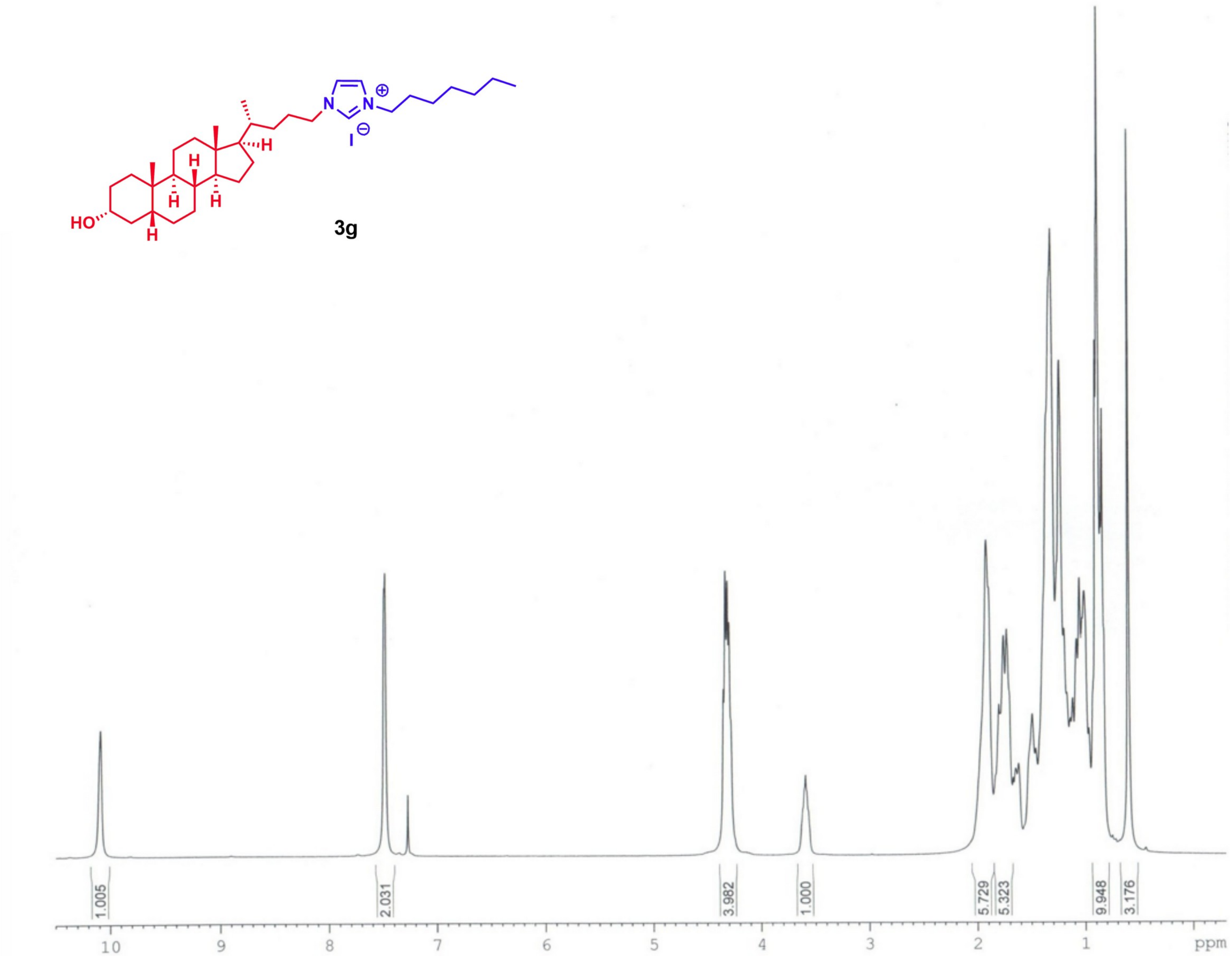

^{13}C NMR spectrum of *N*-heptyl-*N'*-(3α-hydroxy-5β-cholan-24-yl)imidazolium iodide (**3g**)

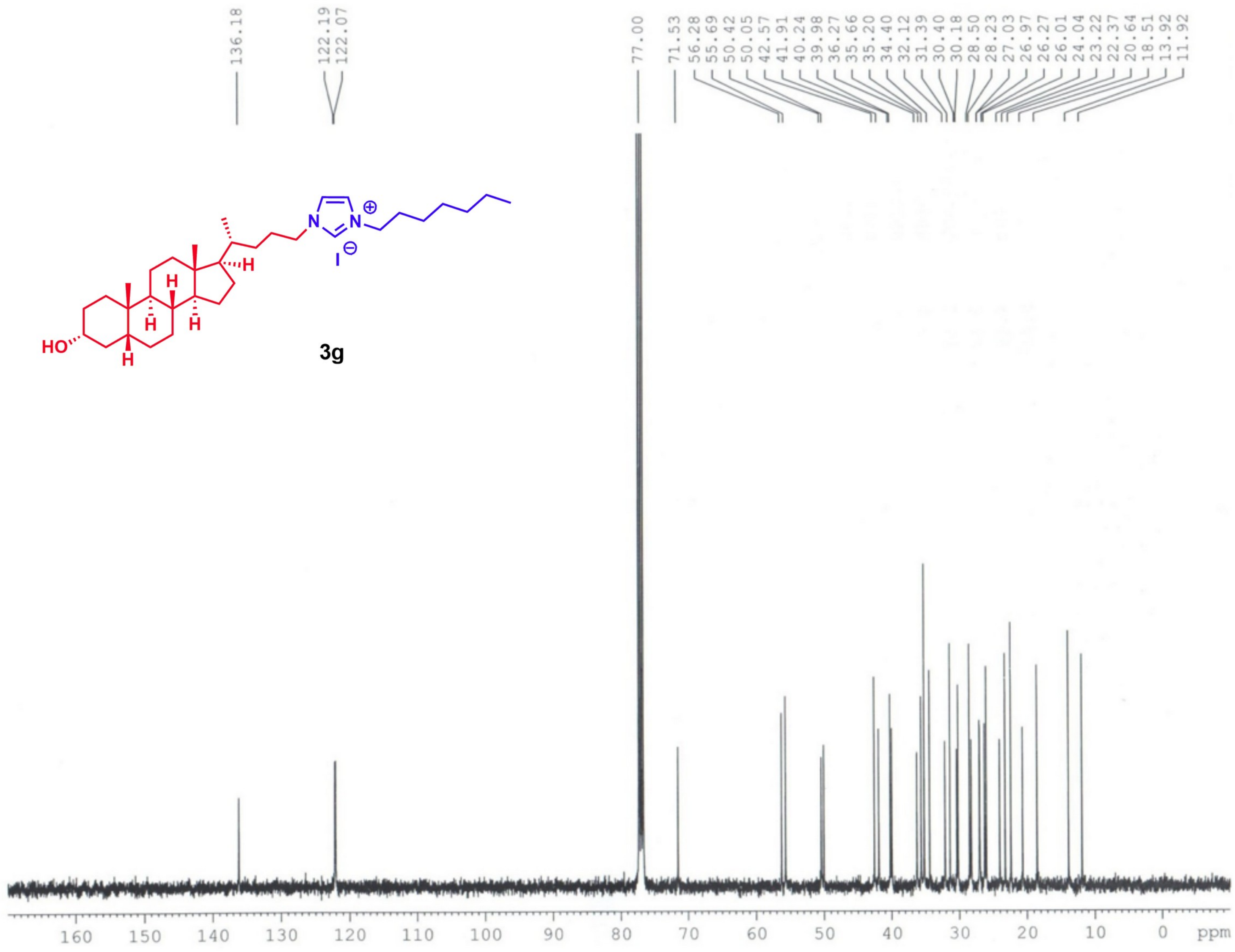

^{1}H NMR spectrum of *N*-(3α-hydroxy-5β-cholan-24-yl)-*N*'-octylimidazolium iodide (**3h**)

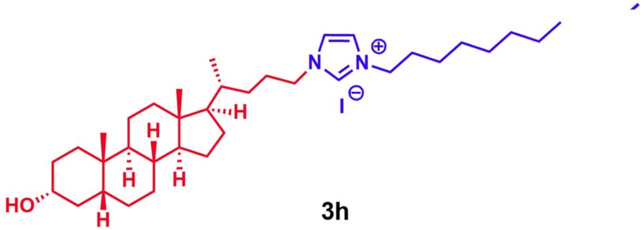

3h

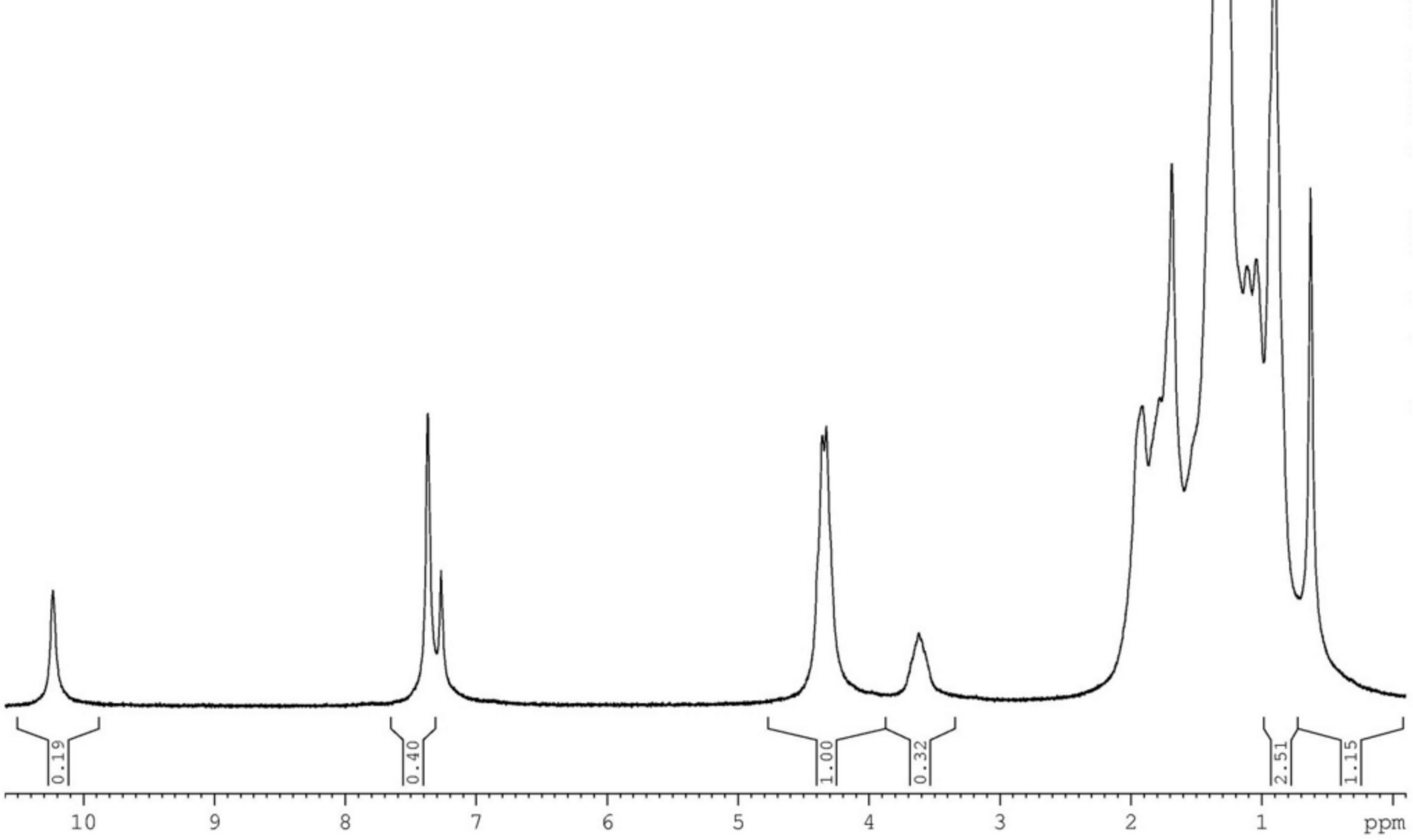

^{13}C NMR spectrum of *N*-(3α-hydroxy-5β-cholan-24-yl)-*N*’-octylimidazolium iodide (**3h**)

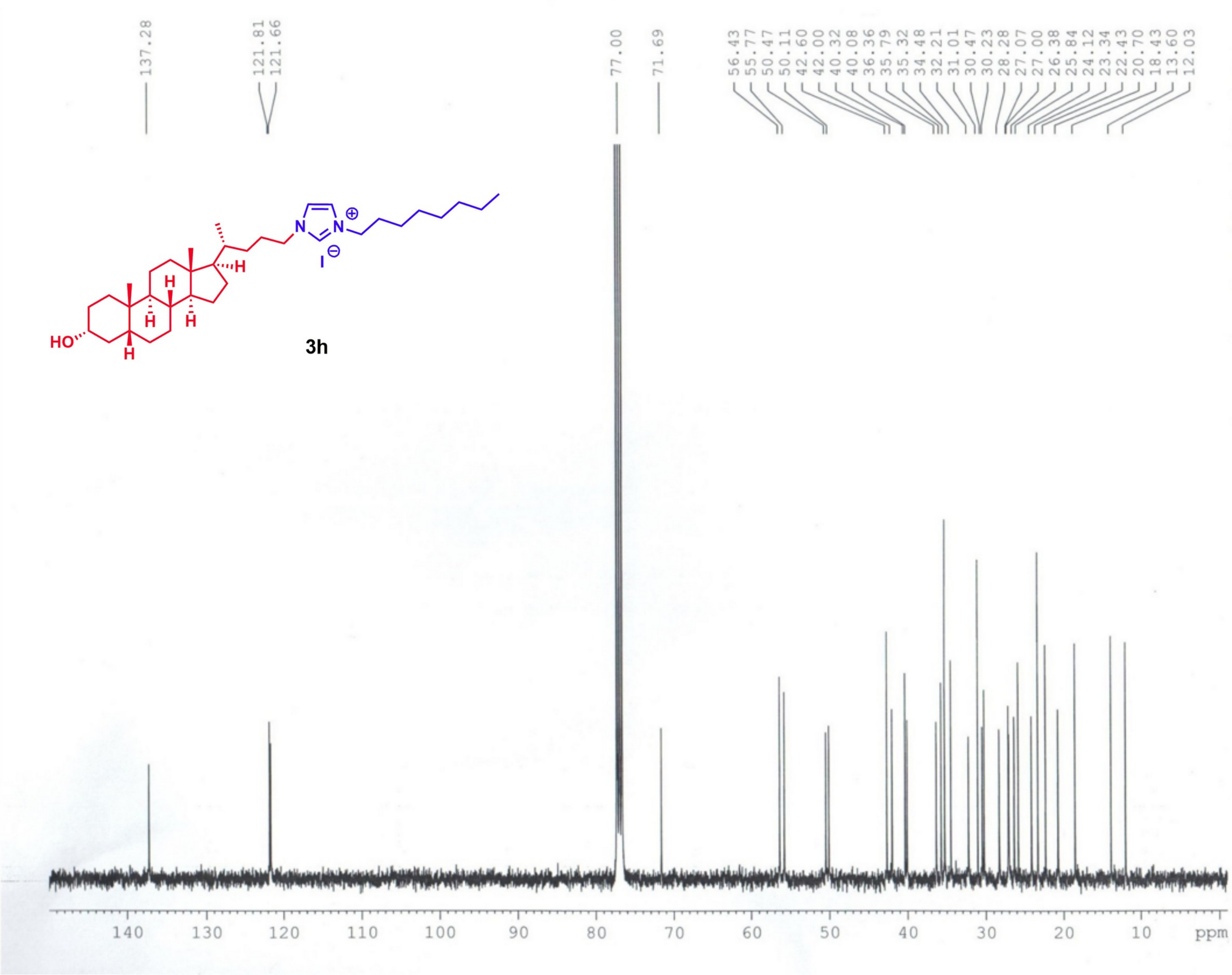

^{1}H NMR spectrum of *N*-dodecyl-*N'*-(3α-hydroxy-5β-cholan-24-yl)imidazolium iodide (**3i**)

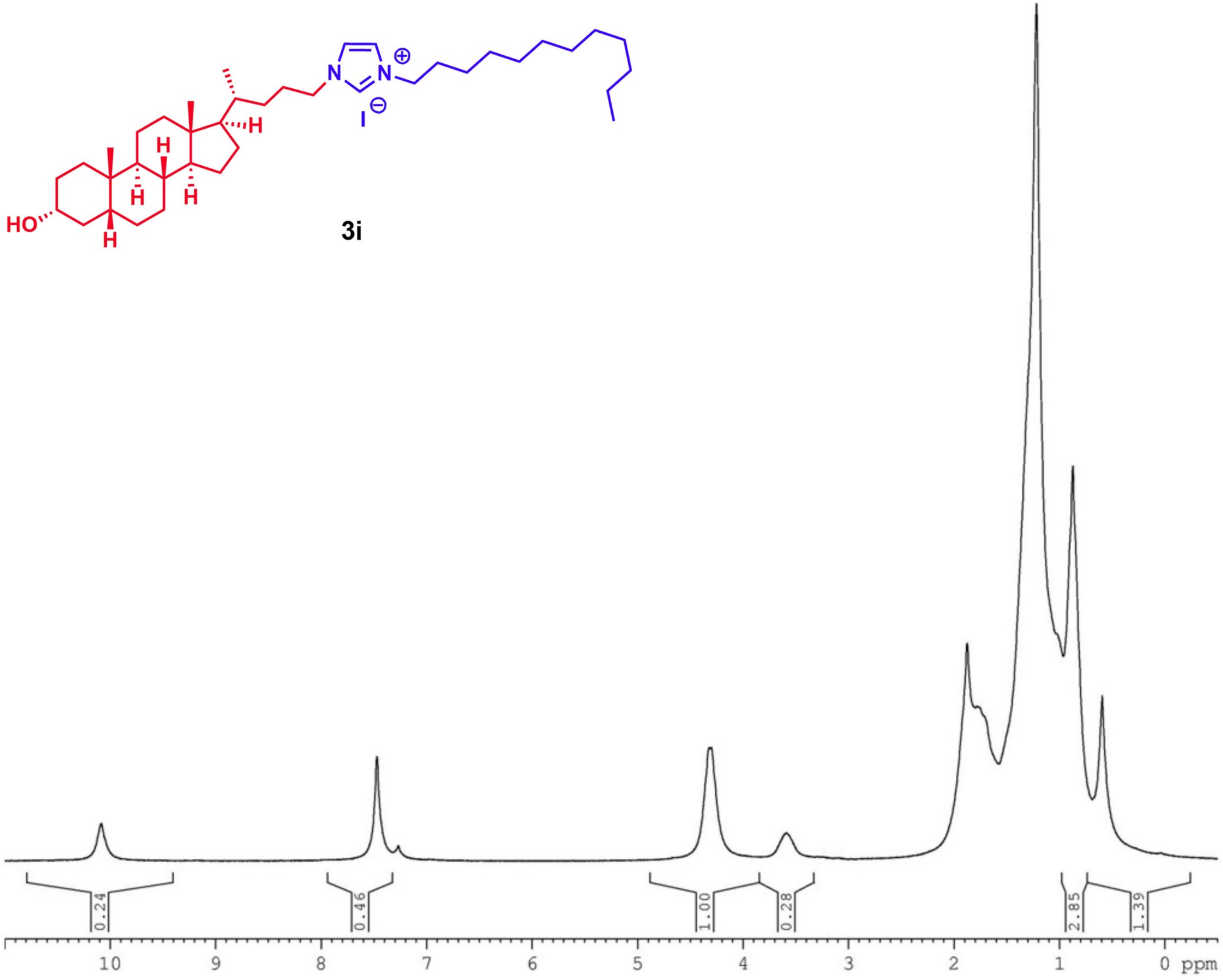

^{13}C NMR spectrum of *N*-dodecyl-*N*'-(3α-hydroxy-5β-cholan-24-yl)imidazolium iodide (**3i**)

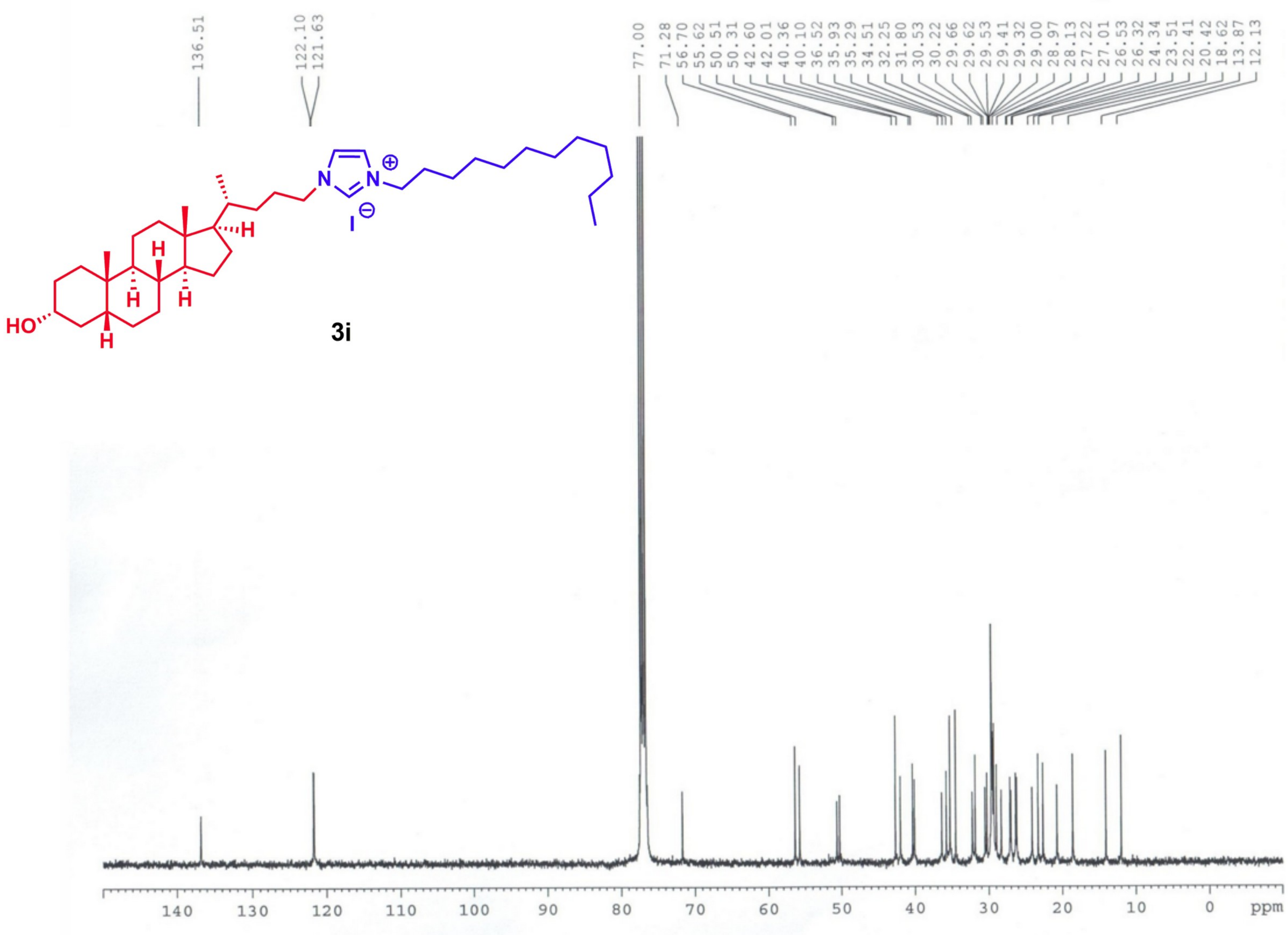

^{1}H NMR spectrum of *N*-hexadecyl-*N*'-(3α-hydroxy-5β-cholan-24-yl)imidazolium iodide (**3j**)

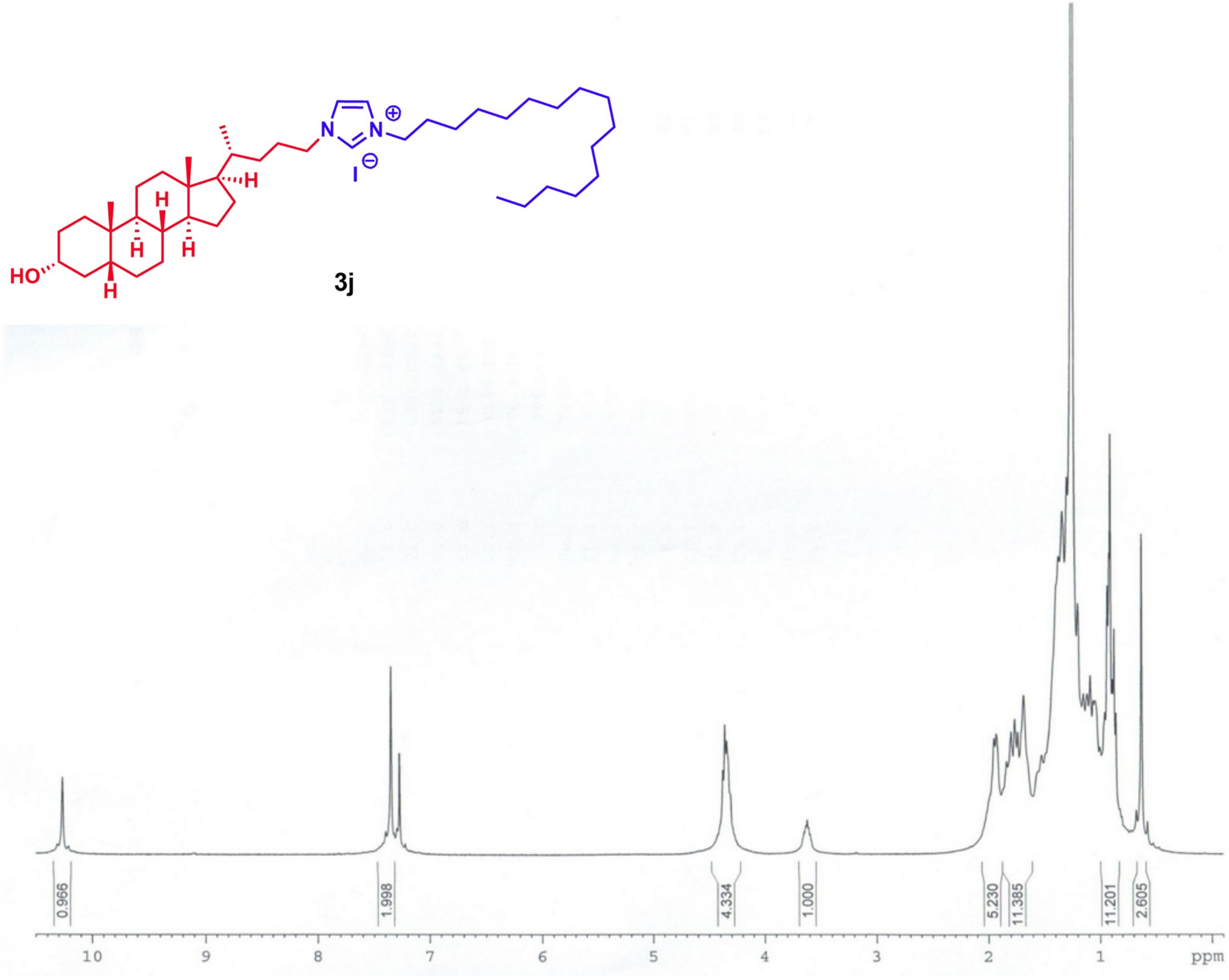

^{13}C NMR spectrum of *N*-hexadecyl-*N'*-(3α-hydroxy-5β-cholan-24-yl)imidazolium iodide (**3j**)

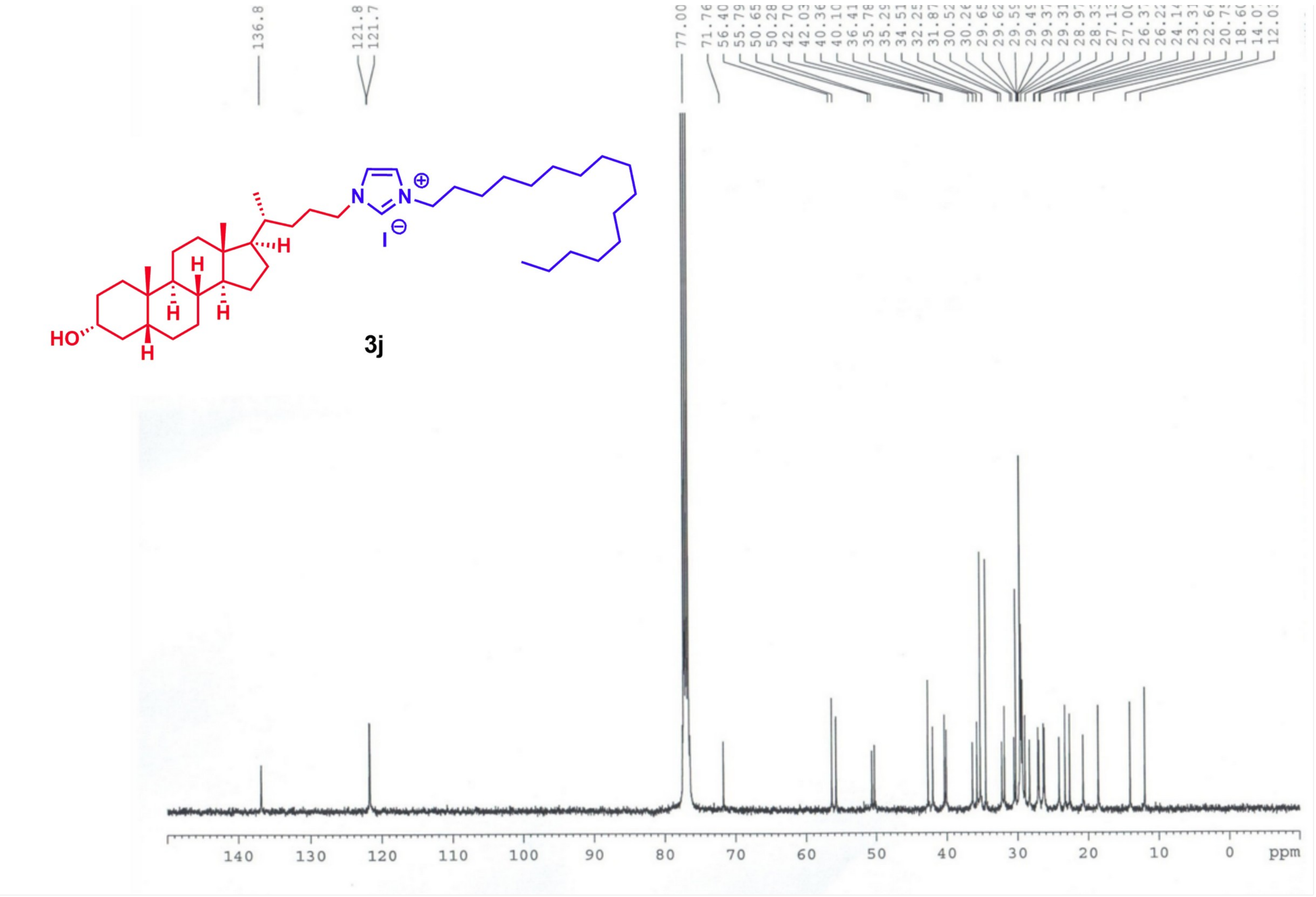

^{1}H NMR spectrum of *N*-(3-oxo-23,24-dinorchol-4-en-22-yl)-*N*'-propylimidazolium iodide (**4c**)

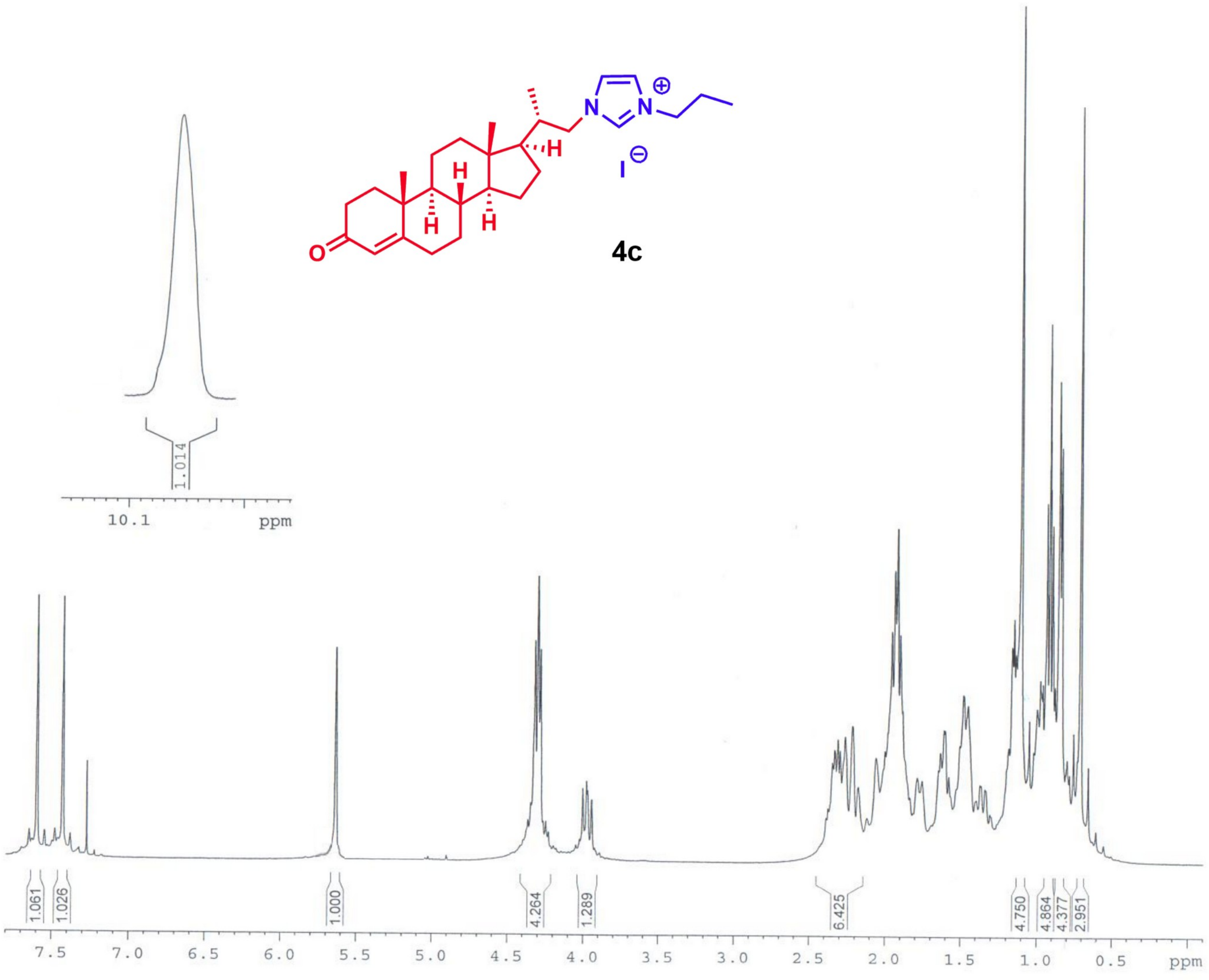

^{13}C NMR spectrum of *N*-(3-oxo-23,24-dinorchol-4-en-22-yl)-*N'*-propylimidazolium iodide (**4c**)

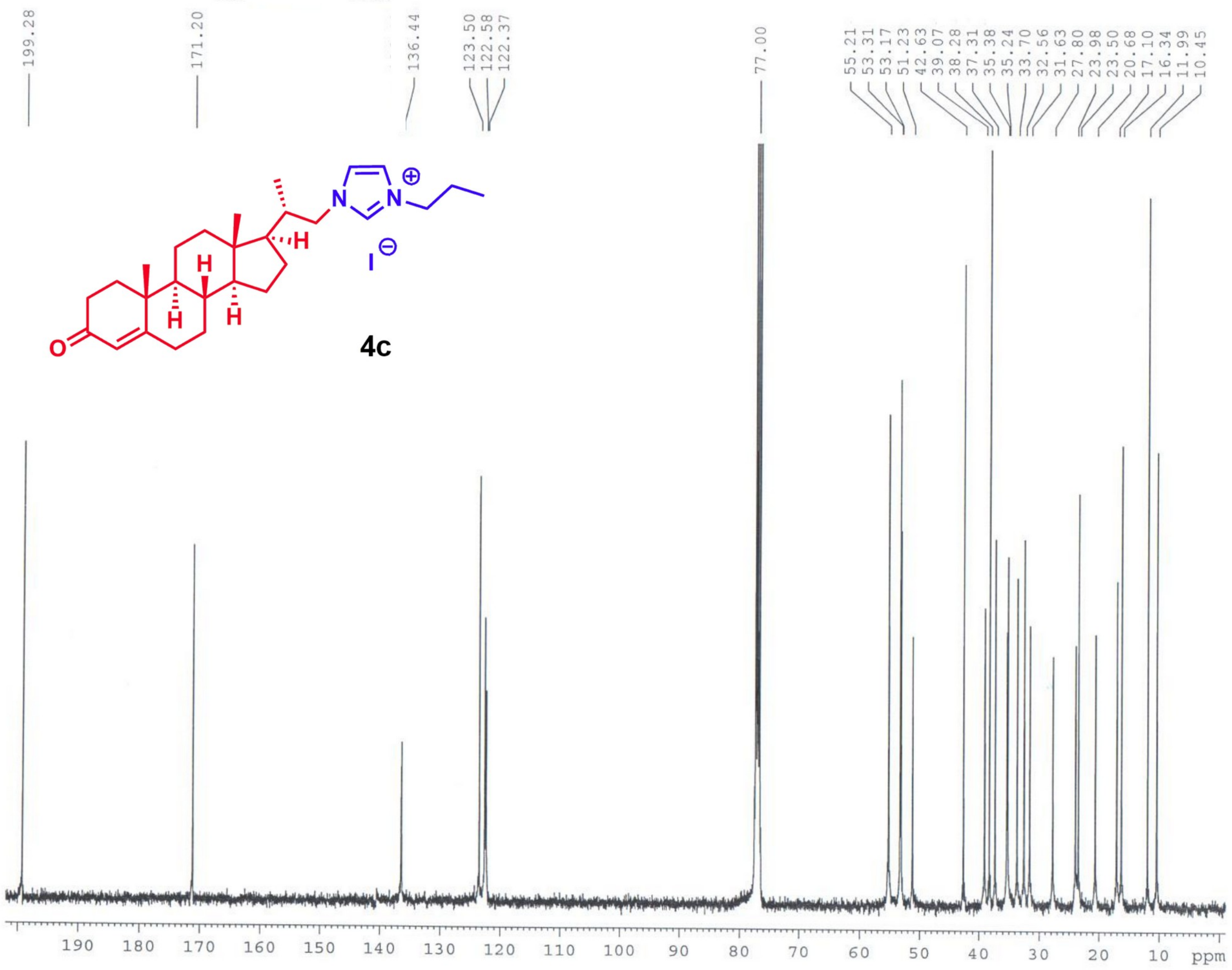

^{1}H NMR spectrum of *N*-butyl-*N'*-(3-oxo-23,24-dinorchol-4-en-22-yl)imidazolium iodide (**4d**)

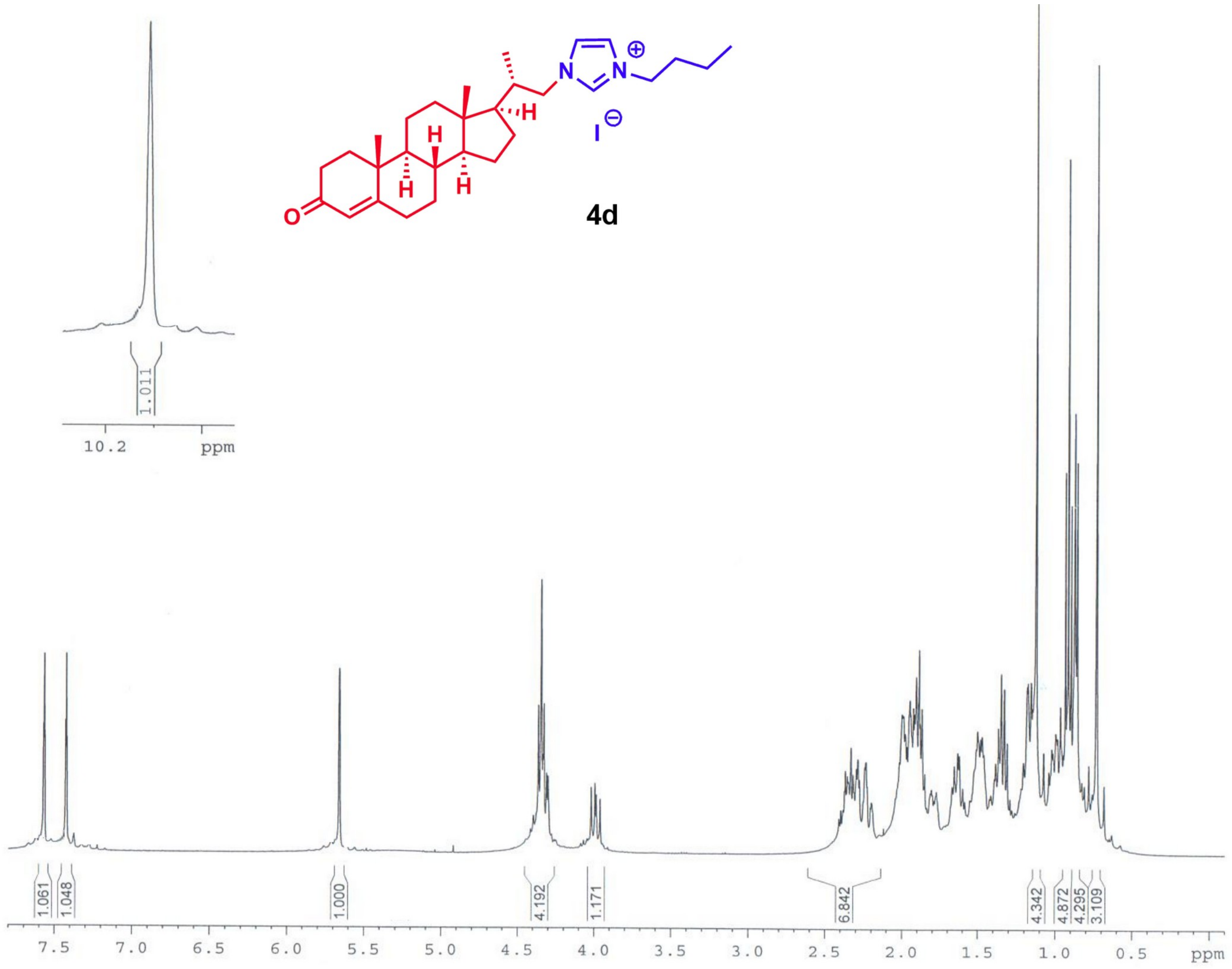

^{13}C NMR spectrum of *N*-butyl-*N'*-(3-oxo-23,24-dinorchol-4-en-22-yl)imidazolium iodide (**4d**)

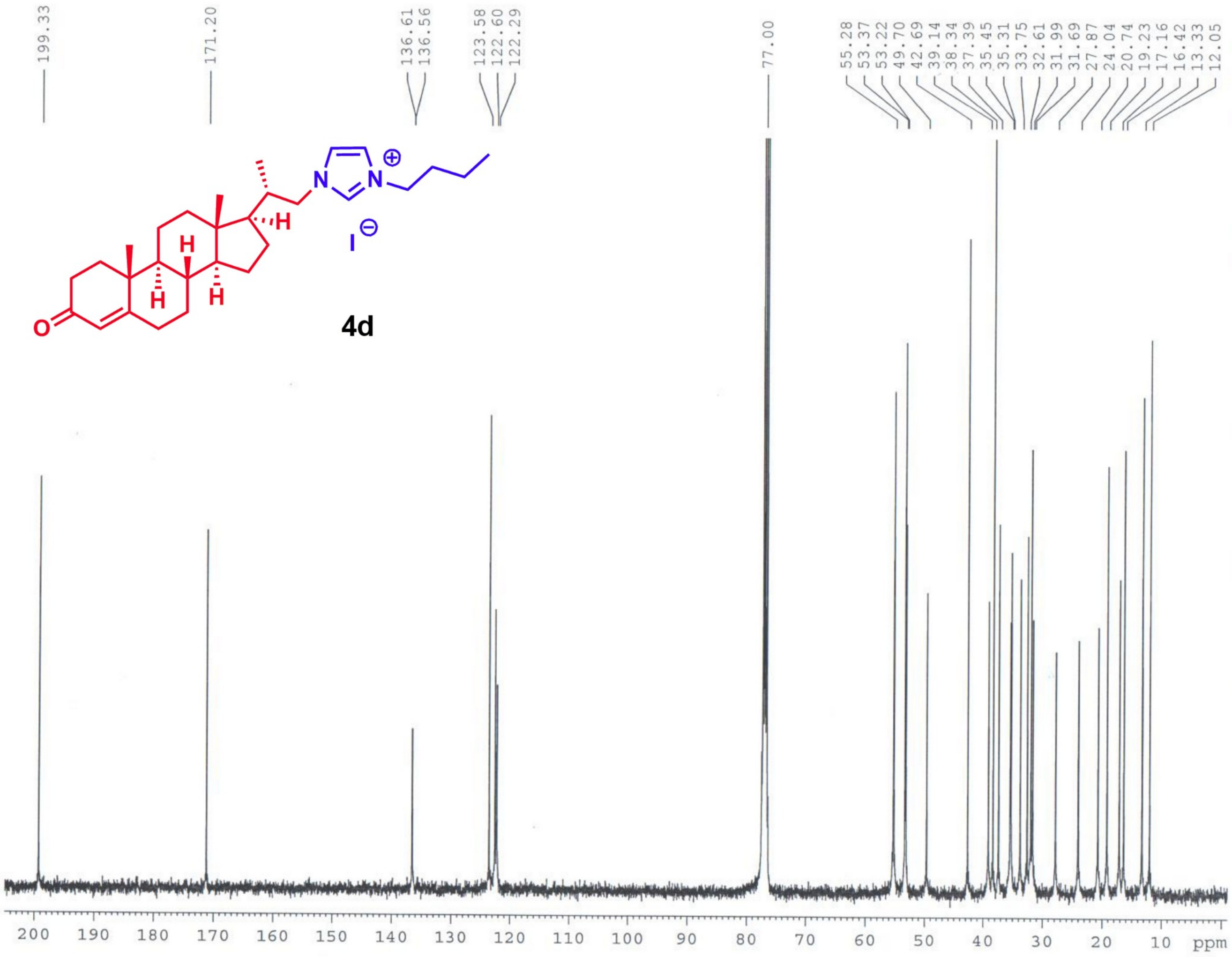

^{1}H NMR spectrum of *N*-heptyl-*N*’-(3-oxo-23,24-dinorchol-4-en-22-yl)imidazolium iodide (**4g**)

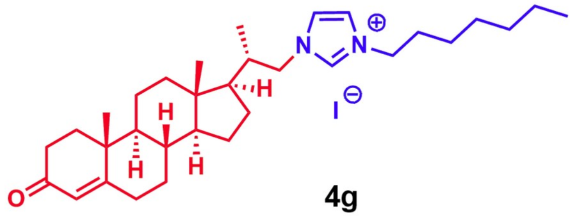

4g

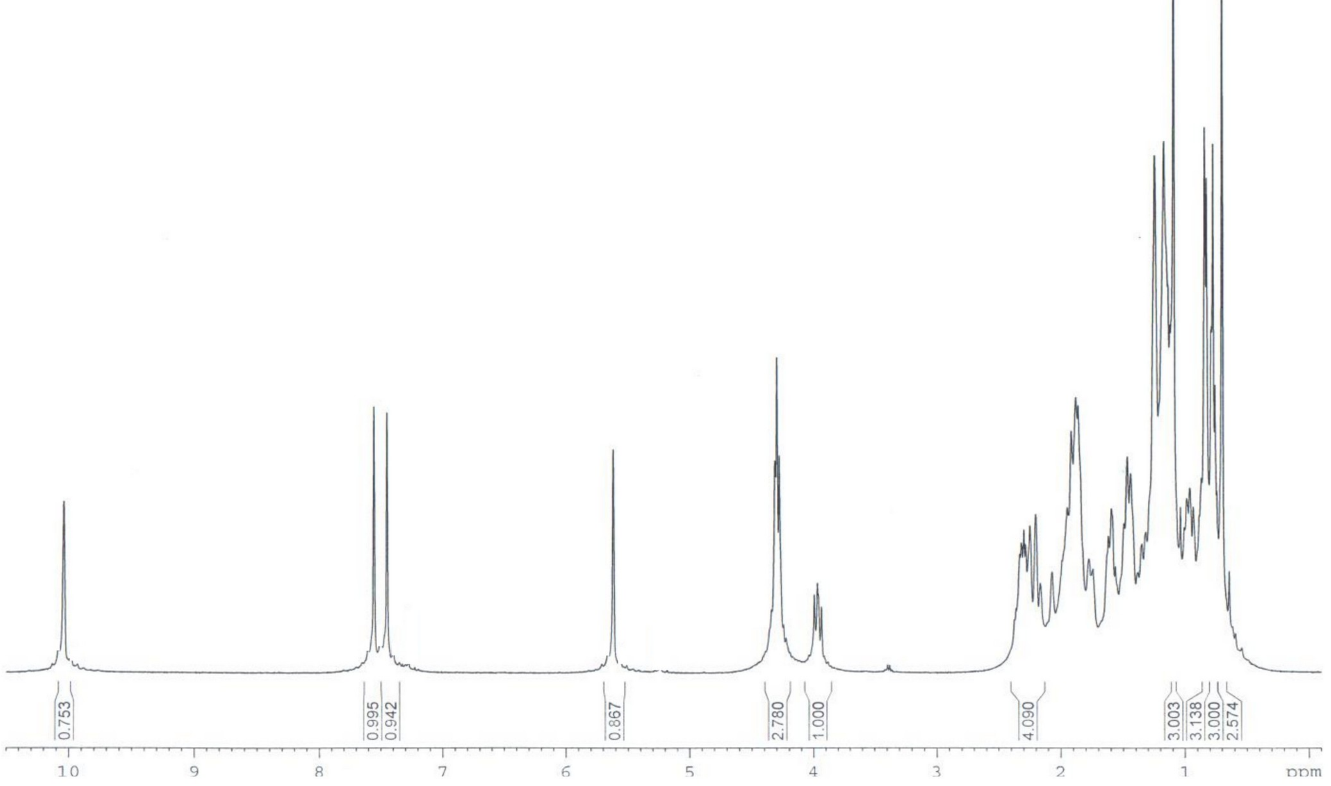

^{13}C NMR spectrum of *N*-heptyl-*N*’-(3-oxo-23,24-dinorchol-4-en-22-yl)imidazolium iodide (**4g**)

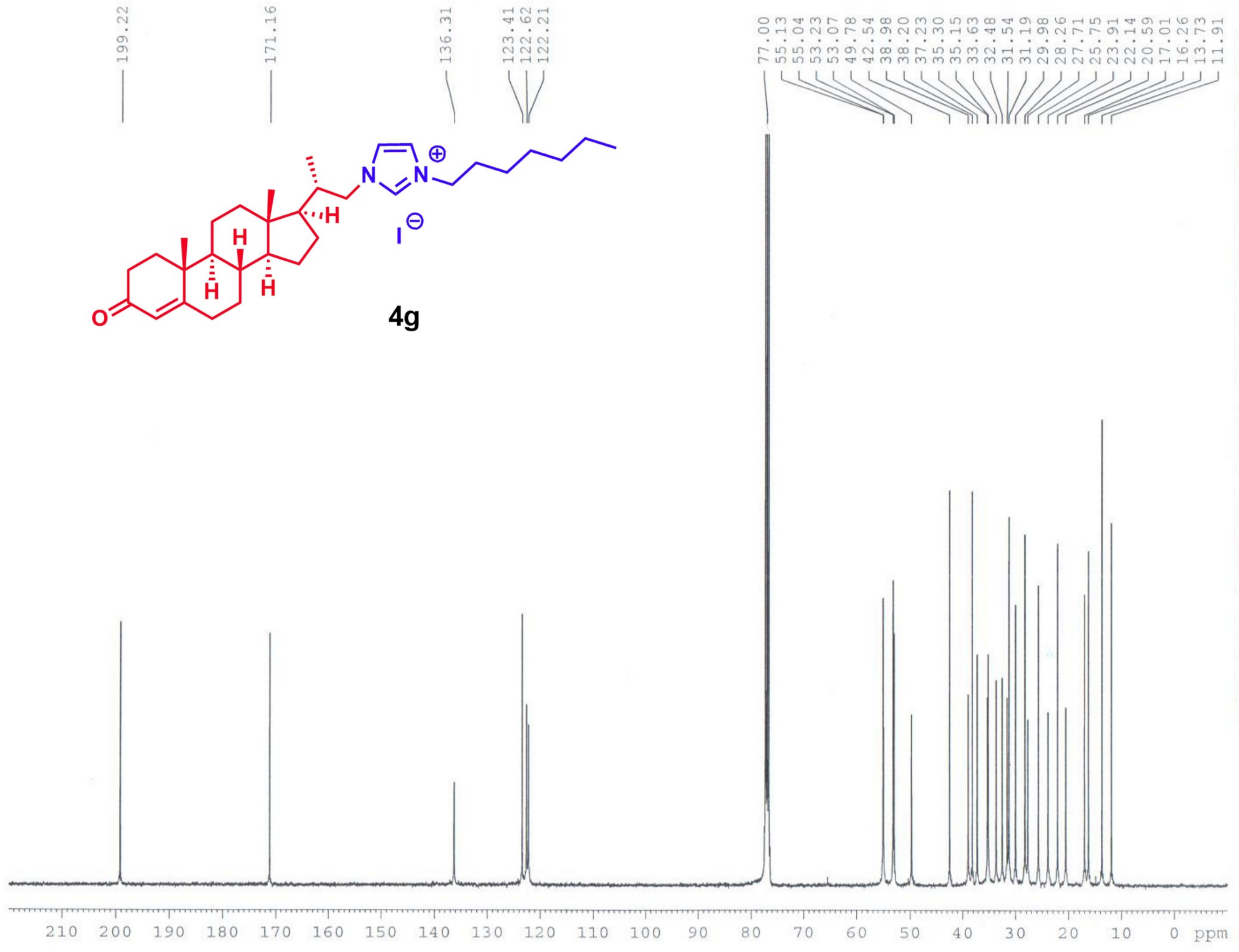

^{1}H NMR spectrum of *N*-octyl-*N'*-(3-oxo-23,24-dinorchol-4-en-22-yl)imidazolium iodide (**4h**)

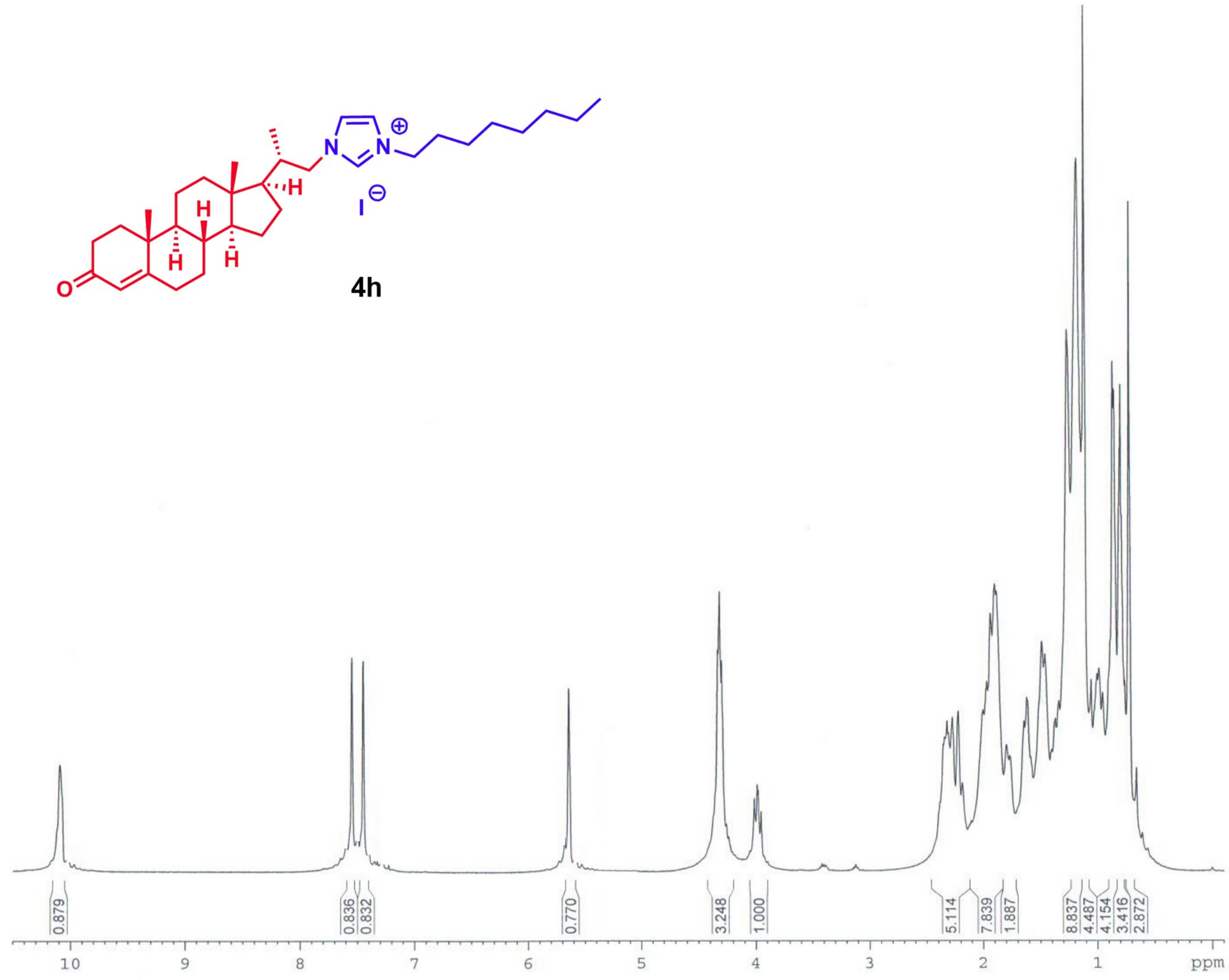

^{13}C NMR spectrum of *N*-octyl-*N'*-(3-oxo-23,24-dinorchol-4-en-22-yl)imidazolium iodide (**4h**)

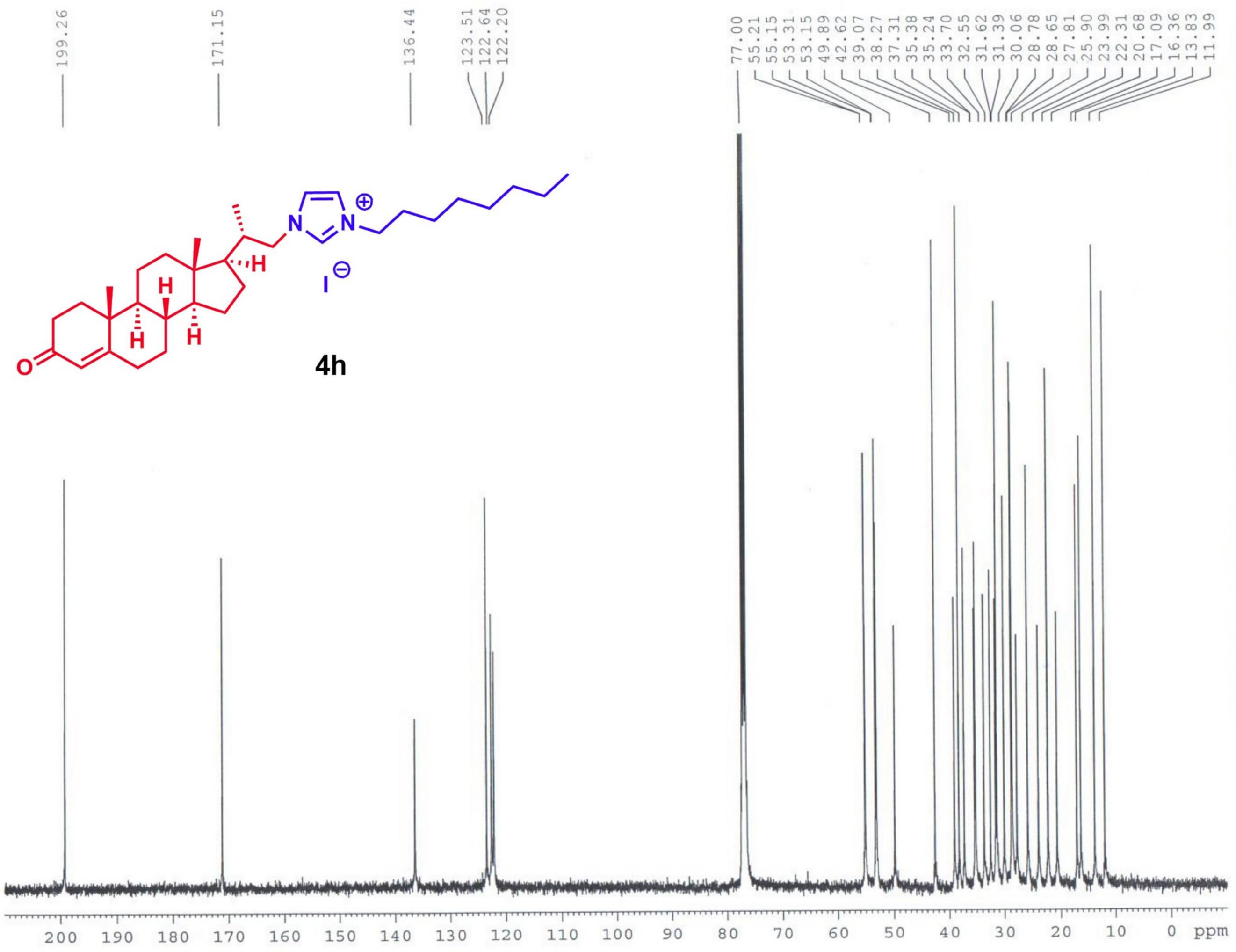

^{1}H NMR spectrum of *N*-dodecyl-*N'*-(3-oxo-23,24-dinorchol-4-en-22-yl)imidazolium iodide (**4i**)

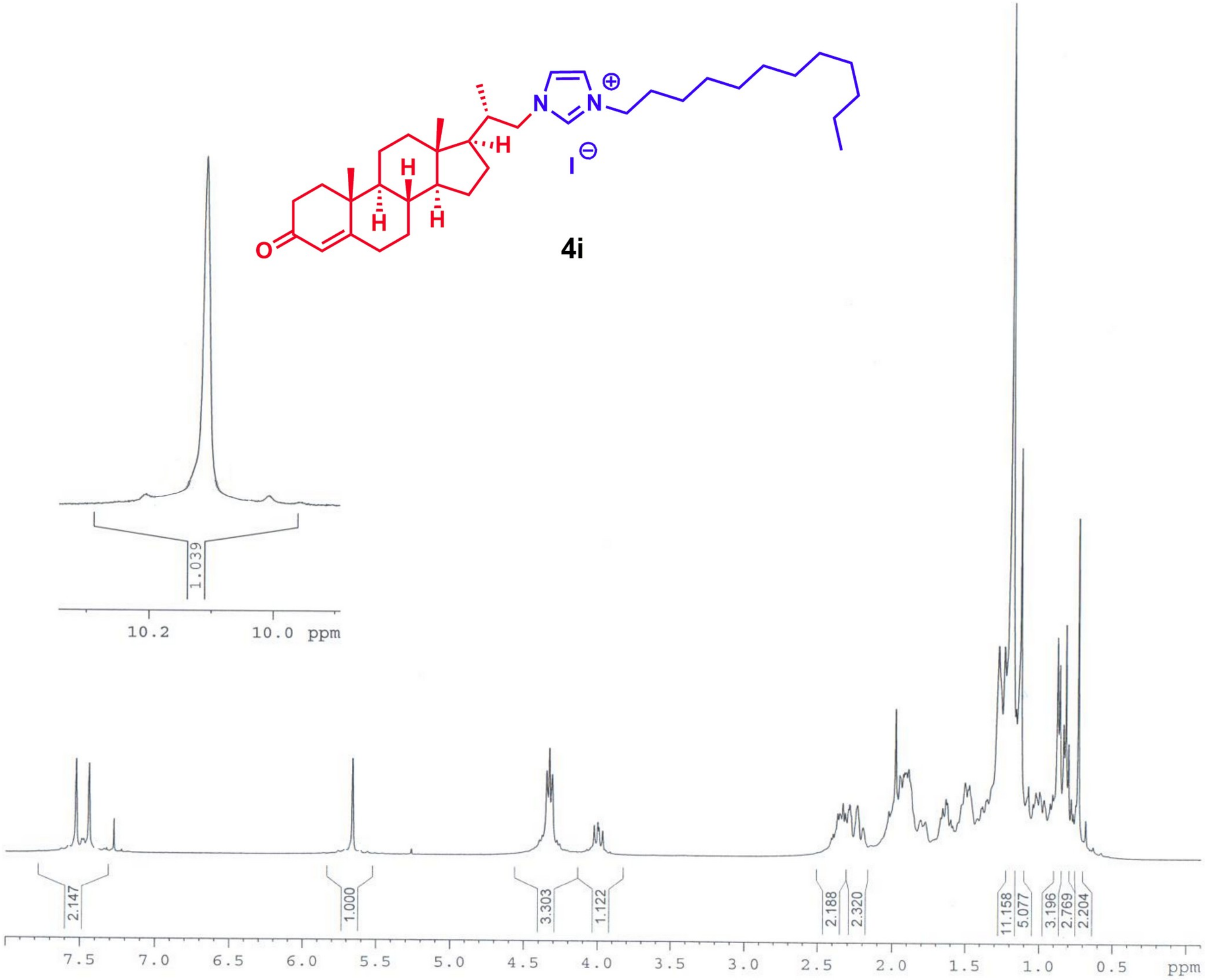

^{13}C NMR spectrum of *N*-dodecyl-*N'*-(3-oxo-23,24-dinorchol-4-en-22-yl)imidazolium iodide (**4i**)

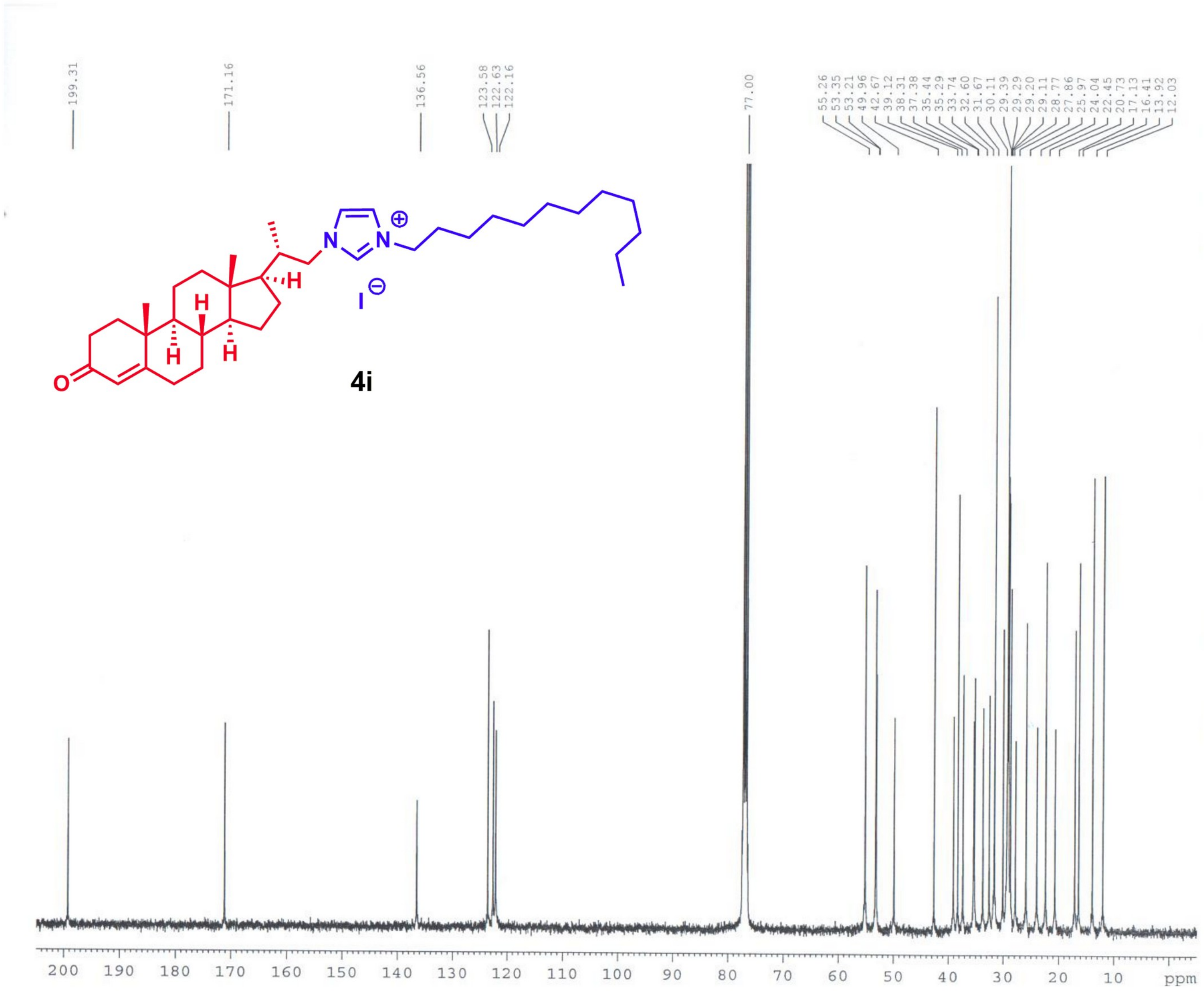

^{1}H NMR spectrum of *N*-hexadecyl-*N*’-(3-oxo-23,24-dinorchol-4-en-22-yl)imidazolium iodide (**4j**)

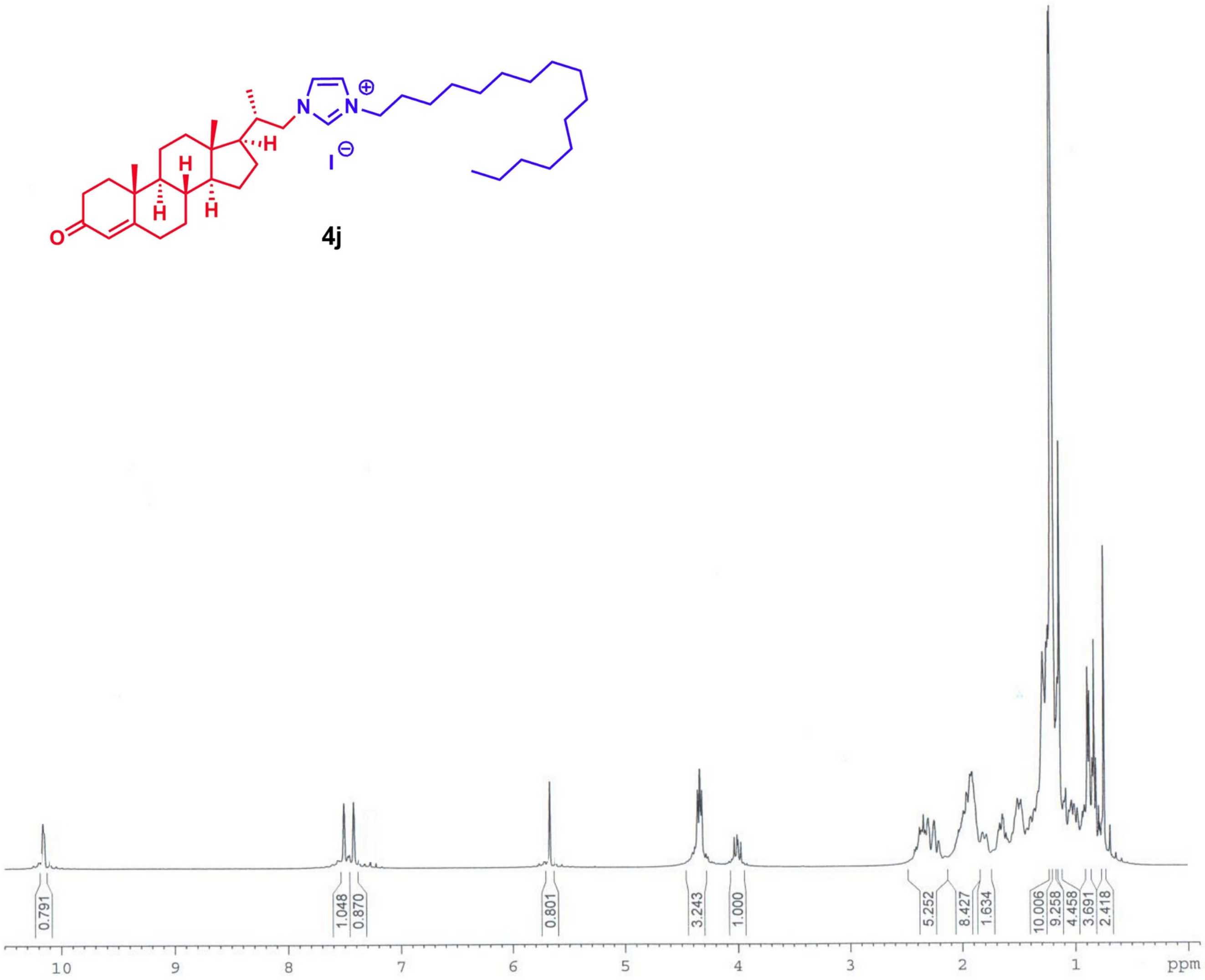

4j

^{13}C NMR spectrum of *N*-hexadecyl-*N'*-(3-oxo-23,24-dinorchol-4-en-22-yl)imidazolium iodide (**4j**)

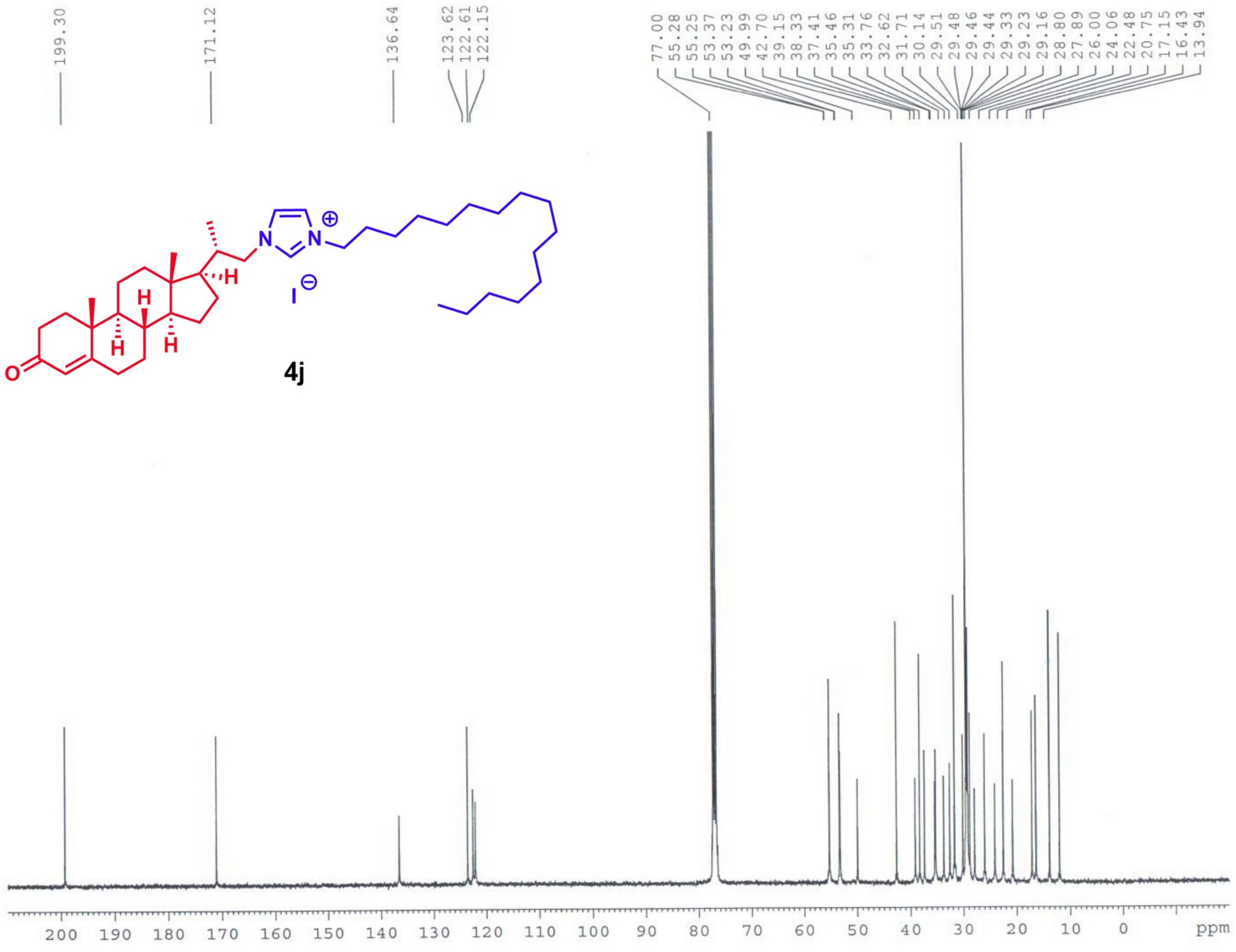

Supplement: Supplementary file 1 [file ijms-22-12180-s001.zip › ijms-1425160-supplementary.pdf]
